# Emerging from the ice‐fungal communities are diverse and dynamic in earliest soil developmental stages of a receding glacier

**DOI:** 10.1111/1462-2920.14598

**Published:** 2019-04-11

**Authors:** Philipp Dresch, Johannes Falbesoner, Chiara Ennemoser, Michaela Hittorf, Regina Kuhnert, Ursula Peintner

**Affiliations:** ^1^ Institute of Microbiology University Innsbruck Innsbruck Austria

## Abstract

We used amplicon sequencing and isolation of fungi from in‐growth mesh bags to identify active fungi in three earliest stages of soil development (SSD) at a glacier forefield (0–3, 9–14, 18–25 years after retreat of glacial ice). Soil organic matter and nutrient concentrations were extremely low, but the fungal diversity was high [220 operational taxonomic units (OTUs)/138 cultivated OTUs]. A clear successional trend was observed along SSDs, and species richness increased with time. Distinct changes in fungal community composition occurred with the advent of vascular plants. Fungal communities of recently deglaciated soil are most distinctive and rather similar to communities typical for cryoconite or ice. This indicates melting water as an important inoculum for native soil. Moreover, distinct seasonal differences were detected in fungal communities. Some fungal taxa, especially of the class Microbotryomycetes, showed a clear preference for winter and early SSD. Our results provide insight into new facets regarding the ecology of fungal taxa, for example, by showing that many fungal taxa might have an alternative, saprobial lifestyle in snow‐covered, as supposed for a few biotrophic plant pathogens of class Pucciniomycetes. The isolated fungi include a high proportion of unknown species, which can be formally described and used for experimental approaches.

## Introduction

Since the last Little Ice Age around 1858, glaciers are receding all over the world (Abermann *et al.,* 2009). Glacier forefields provide valuable areas for studying the development and colonization of newly exposed soils. Micro‐organisms are the first life forms to colonize freshly exposed substrates and play a tremendous role in soil formation (Sigler *et al.,* 2002; Sigler and Zeyer, 2002; Sigler and Zeyer, 2004; Nicol *et al.,* 2005; Bardgett *et al.,* 2007; Nemergut *et al.,* 2007; Schmidt *et al.,* 2008; Ciccazzo *et al.,* 2016). Biological soil crusts consist of typical pioneer organisms (bacteria including cyanobacteria, fungi, algae and lichens) colonizing the soil surface and subsurface. They perform a key role in stabilizing mobile surfaces, thus protecting soil from erosion and cryoturbation (Bu *et al.,* 2015). Biological soil crust productivity plays an important role in the carbon cycle during the early stage of succession: In a high Arctic glacier foreland, the estimated biological soil crust net primary production was higher than the net primary production of the other vegetation (Yoshitake *et al.,* 2009). There is a mutual influence between microbes and the environment they colonize. Soil microbial community (SMC) composition and thus primary succession is strongly influenced by soil development in the glacier forefield (Jumpponen, 2003; Tscherko *et al.,* 2005), and especially, active fungal community composition changes over soil developmental stages (Rime *et al.,* 2016a; Rime *et al.,* 2016b).

SMCs follow distinct seasonal patterns across different habitats including boreal forest and arctic/alpine habitats (Schadt *et al.,* 2003; Kuhnert *et al.,* 2012; Lazzaro *et al.,* 2012; Buckeridge *et al.,* 2013; Koranda *et al.,* 2013; Voříšková *et al.,* 2014; Lazzaro *et al.,* 2015; Schostag *et al.,* 2015; Zifcakova *et al.,* 2016) and are not at all dormant during winter. A community shift from bacteria towards cold‐adapted fungi has been observed during winter (Schadt *et al.,* 2003; Lazzaro *et al.,* 2012; Buckeridge *et al.,* 2013), with saprobial Ascomycota and basidiomycete yeasts‐dominating fungal communities (Oberkofler and Peintner, 2008; Kuhnert *et al.,* 2012). These winter‐active decomposers respire considerable amounts of CO_2_ (Schmidt and Lipson, 2004; Monson *et al.,* 2006; Zinger *et al.,* 2009; Aanderud *et al.,* 2013) and build up about 10 times more biomass during winter than during summer (Kuhnert *et al.,* 2012), thus being main players for soil organic matter (SOM) formation and soil development in successional sites.

New fungal lineages discovered from snow‐covered habitats (Schadt *et al.,* 2003; Porter *et al.,* 2008) boosted the scientist's interest in SMCs of cold habitats. This resulted in the description of many new fungal taxa, like snow chytrids (Naff *et al.,* 2013), psychrophilic yeasts (Margesin and Fell, 2008; Butinar *et al.,* 2011) and cold‐tolerant filamentous fungi (Wang *et al.,* 2015; Peintner *et al.,* 2016). Novel fungal taxa associated with snow or cryoconite were because continuously detected (e.g., Edwards *et al.,* 2013; Brown and Jumpponen, 2014), indicating that snow‐covered soil also harbours a high diversity of unknown fungal taxa.

In this work, we focus on the earliest 25 years of soil development of a retreating glacier to identify first fungal colonizers and investigate how long it takes to develop a stable community. In this context, we especially address fungi actively growing underneath the snow cover by using a combination of in‐growth mesh bags (MBs) (Wallander *et al.,* 2001) with both, cultivation as well as amplicon sequencing. This combination of approaches provides reliable information on seasonal variation in active fungal communities at three stages of soil development (SSD) within our 25‐year chronosequence. The obtained NGS data and fungal isolates shall provide a solid base for future studies on the diversity of cold‐adapted fungi.

## Results

### *SOM content is extremely low and increases within 25 years of deglaciation*

**Figure 1:**
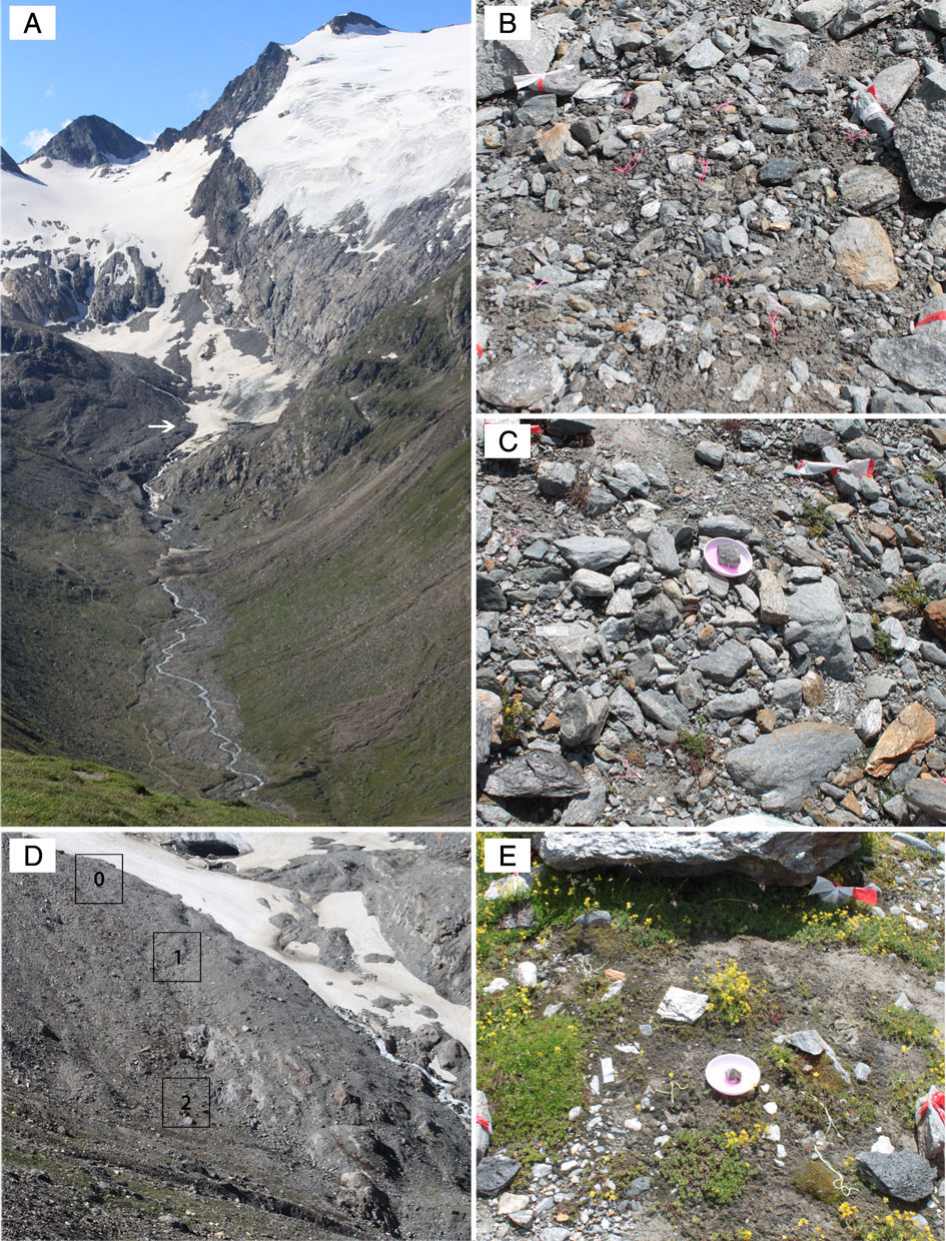
*A*–*E* Sampling sites at the Rotmoosferner glacier forefield at 2280–2450 m above sea level. *A*. Rotmoosferner glacier forefield, the arrow is indicating the sampling area close to the glacier tongue. *B*. Representative sampling plot of site 0 (0–3 years ice free). *C*. Sampling plot of site 1 (7–14 years ice free). *D*. Overview of the sampling area, black frames indicate the sampling sites with different stages of soil development ranging from 0 to 25 years (site 0, site 1 and site 2). *E*. Sampling plot of site 2 (18–25 years ice free). [Color figure can be viewed at http://wileyonlinelibrary.com]

Sampling was carried out close to the glacier tongue in three earliest stages deglaciation ranging from 0 to 25 years (Fig. 1). Within these first 25 years of soil development, soil pH was neutral (7.5) in all sites due to the relatively high carbonate content, which increased along SSDs (Supporting Information Table S1). SOM was extremely low in all sites, ranging from 0.3% to 0.9% and increased significantly with successional age. Ammonium, nitrate and phosphate also increased. Availability of ammonium and phosphate was significantly correlated with SOM content. Furthermore, ammonium concentrations were significantly correlated to soil water content (Supporting Information Table S2).

### *Diversity of fungi isolated from in‐growth MBs is high, with high proportions of fungi with seasonal preferences and of psychrophilic fungi*

**Figure 2:**
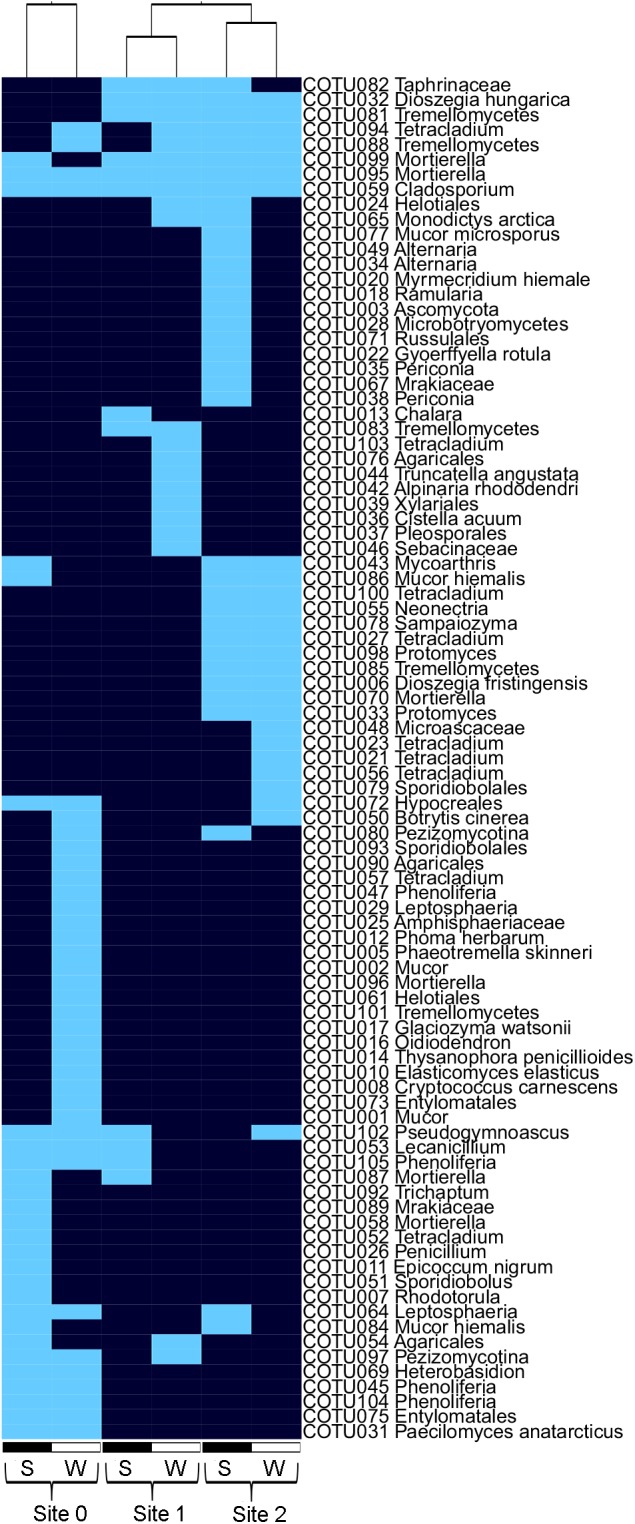
The presence/absence of fungal cOTUs (97% ITS sequence identity) isolated from in‐growth mesh bags, which were buried during the snow‐free vegetation period (summer = S) or the snow‐covered period (winter = W), respectively. The three early stages of soil development are site 0 (0–3 years), site 1 (7–14 years) and site 2 (18–25 years). Seasonal patterns are especially pronounced during the first 0–3 years of soil development. [Color figure can be viewed at http://wileyonlinelibrary.com]

In total, 138 fungal cOTUs (cultivated strains merged into operational taxonomic units) with 99% sequence similarity were isolated from the MBs. When using a threshold of 97%, several cOTUs collapsed, resulting in 91 cOTUs total; of these, 89 contained a full ITS2 region and were used for comparison with amplicon sequencing data. More than half (54%) of the isolated fungi were Ascomycota, about one‐third (34%) Basidiomycota, 7% Mortierellomycota and 5% Mucoromycota. On the genus level, 12% of the fungal isolates could not be identified (Supporting Information Table S3). The most species‐rich genera were *Tetracladium* (7 cOTUs), *Mortierella* (6 cOTUs), *Mucor* (5 cOTUs) and *Phenoliferia ss. lato* (10 cOTUs). Yeasts made up of 30% of the isolated fungi (single‐celled, budding fungi; 41 cOTUs). More than half of the isolated cOTUs (*n* = 56) showed seasonal preferences, as they were either isolated from winter‐MBs (*n* = 33) or summer‐MBs (*n* = 23), only (Fig. 2). Furthermore, the proportion of psychrophilic fungi (no growth at 25°C) was generally high (44%, *n* = 40 cOTUs). The number of cOTUs obtained was highest in the earliest SSD (50 OTUs in site 0 compared to 26 OTUs in site 1 and 44 OTUs in site 2). Some fungal isolates showed preferences for specific SSDs: 35% (*n* = 32) were found only in barren ground of site 0, 11% (*n* = 10) only in site 1 and 29% (*n* = 26) exclusively in site 2.

### *Amplicon sequencing confirms that fungal diversity increases with time because deglaciation, but most species remain unidentified*

**Figure 3:**
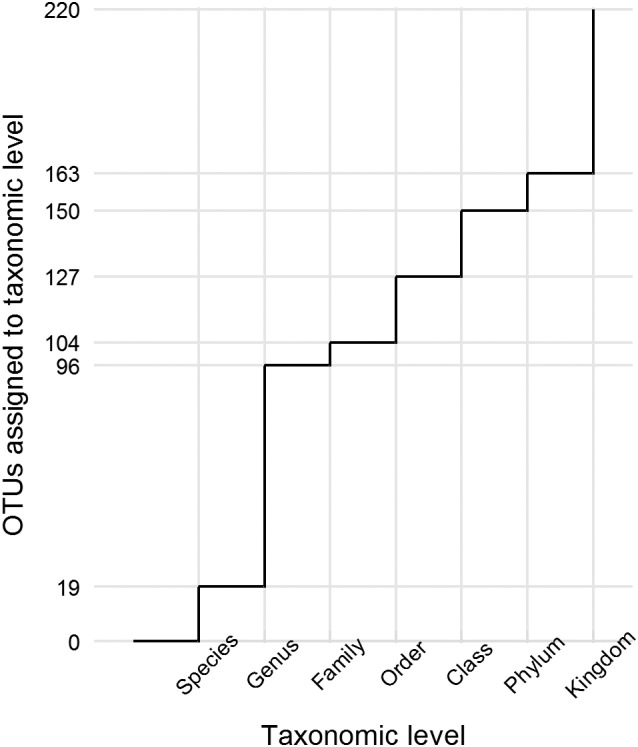
Cumulative step function on amplicon data: number of OTUs identified at a specific taxonomic level (total number of OTUs = 220) from the first 25 years of soil development after deglaciation. The proportion of OTUs, which could not be identified on species level, is very high.

Of all forward and reverse reads (*n* = 937,459), 84.8% passed paired‐end joining and initial quality‐filtering and 67.7% were retained after extraction of fungal ITS2 and disposal of non‐fungal sequences as proposed by ITSx (ver. 1.0.11) (Bengtsson‐Palme *et al.,* 2013). Sequencing depth per sample spanned from 1821 to 45,280 reads with an average of 15,610 ± 9352 SD. The rarefaction to a minimum of 4020 reads per sample led to the exclusion of soil sample B19 (SSD 2). The rarefied data set consisted of 938 OTUs. Removing low abundance and low prevalence OTUs reduced the total number of OTUs to 220, of which 40.5% could not be assigned a phylum level taxonomy. The inclusion of manual NCBI BLASTn results (included in all downstream analyses) reduced the fraction of OTUs unidentified at phylum level to 26.4% (Fig. 3). The majority of OTUs was assigned to Ascomycota (39.5%), followed by Basidiomycota (22.3%), Chytridiomycota (5.0%), Mortierellomycota (4.5%) and a low prevalence of other phyla: Rozellomycota, Monoblepharidomycota and Glomeromycota (2.3% in total). On the genus level, 56.4% of all OTUs could not be assigned any taxonomic identification (78.0% without additional NCBI information).

**Figure 4:**
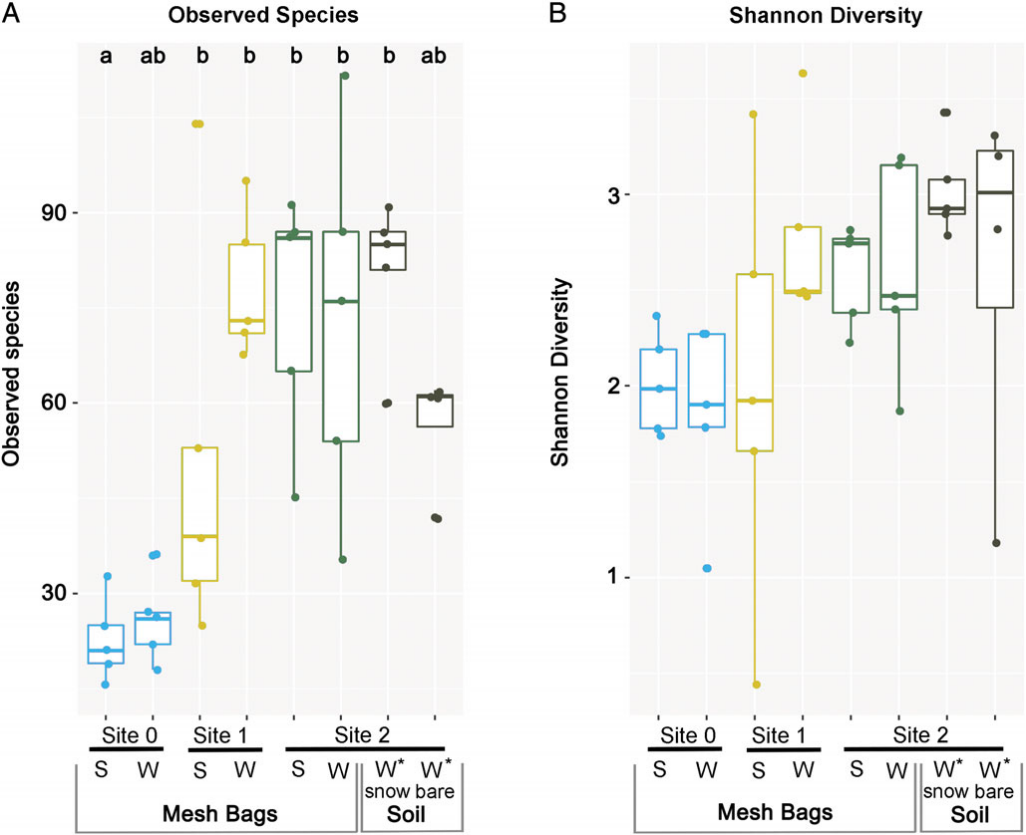
*A,B. Alpha* diversity of fungal communities for the summer (S) and winter (W) period at the three earliest stages of soil development of the glacier forefield based on NGS data of in‐growth mesh‐bag (MB) samples. In addition, a methodological comparison is carried out for winter samples from site 2: MB samples are compared to two types of soil samples: with snow cover (snow) and immediately after snow melt (bare). Asterisks indicate that data are from soil samples. *A*. Observed species diversity. *B*. Shannon diversity. Dots are representing single samples. Site 0 = 0–4 years, site 1 = 9–14 years, site 2 = 18–25 years after deglaciation. Significant differences were detected for the observed species richness, but not for Shannon diversity. Bars with the same letter do not differ significantly (ANOVA, Tukey‐HSD). [Color figure can be viewed at http://wileyonlinelibrary.com]

Alpha‐diversity (Shannon, observed) showed a general increase of fungal richness from the sites closest to the glacier tongue towards older SSDs (Fig. 4). No significant differences in alpha diversity between the two contrasting seasons were found in any of the three SSDs (ANOVA, Tukey‐HSD). Soil samples did not differ significantly from MB samples (*p* = 0.997), and soil samples retrieved about 2 weeks after snowmelt did not show significant differences compared to those obtained from beneath snow cover (*p* = 0.534).

### *Fungal community composition is not different between MB and soil samples, and community shifts at snow‐melt take longer than 2 weeks*

**Figure 5:**
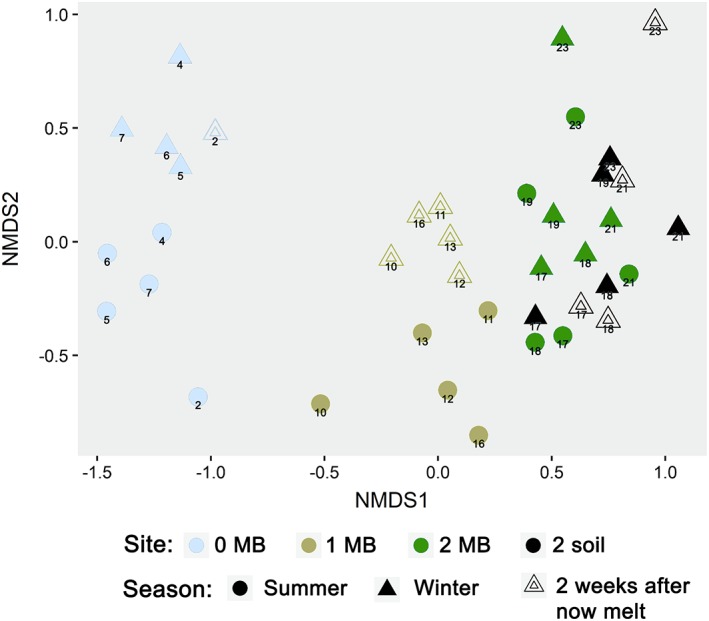
Non‐metric multidimensional scaling (NMDS) plot of fungal communities detected in mesh bags (MB) and soil samples. NMDS is based on a Bray–Curtis dissimilarity matrix of the rarefied, filtered and square‐root transformed OTU abundance data (NGS data). Stress = 0.157. Colours indicate site 0 (lilac), site 1 (green), site 2 (blue). Soil samples were investigated in site 2 during winter only and are represented by pale blue colours. Circles represent summer samples, full triangles winter samples under snow‐cover and nested triangles winter samples retrieved 2 weeks after now‐melt. Fungal communities occurring during the first 3 years of soil development most are distinct and seasonally different. [Color figure can be viewed at http://wileyonlinelibrary.com]

No significant differences between the fungal communities were found with the two different sampling strategies used in this study (MBs vs. soil samples; cf. results of ANOSIM and PERMANOVA in Supporting Information Table S4). Furthermore, soil samples clustered relatively close to the corresponding MB samples in ordination plots (Fig. 5).

**Figure 6:**
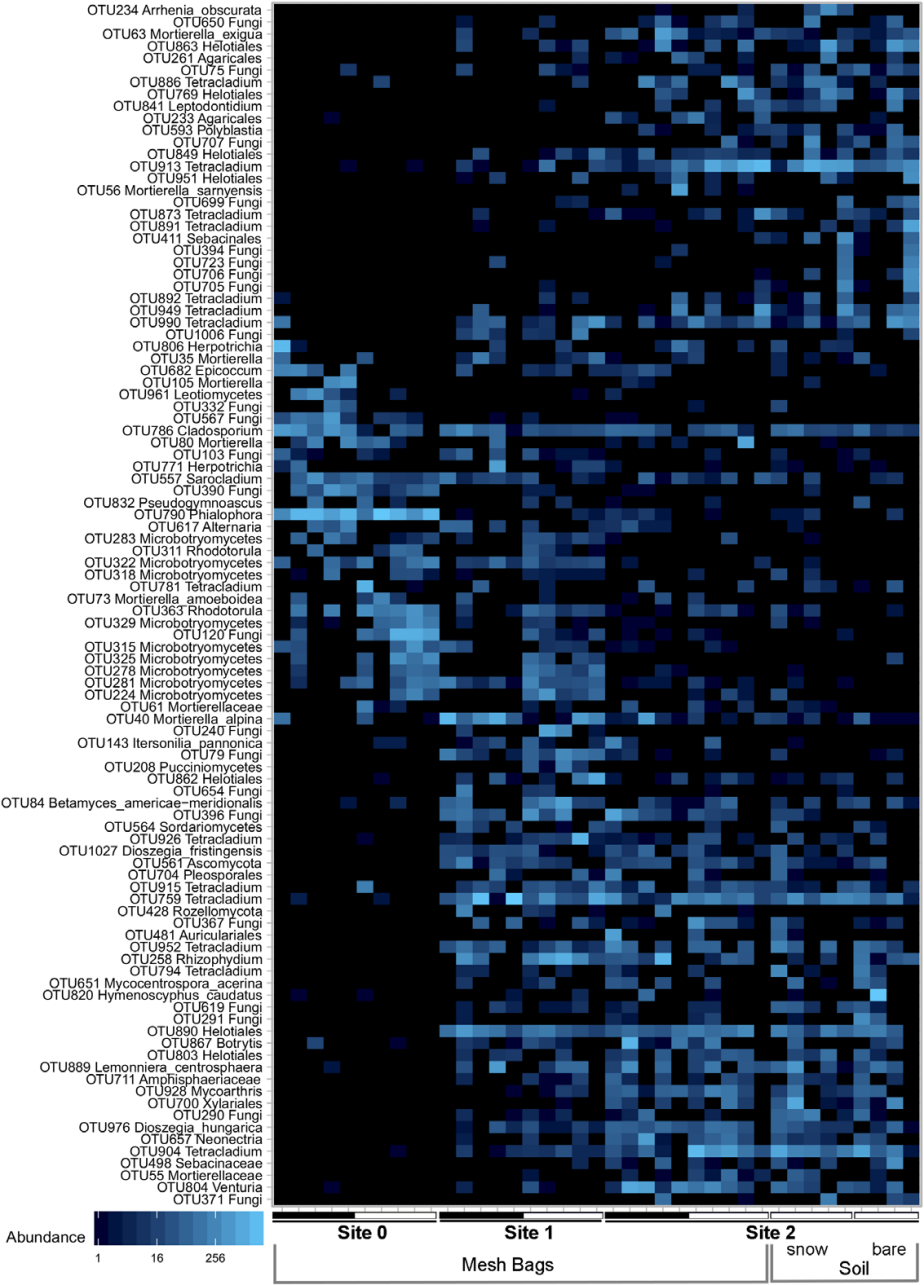
Ordination‐based (NMDS) heat map of 100 most abundant OTUs detected by amplicon sequencing. Data show a clear separation of samples between stages of soil development (site 0: 0–3 years, site 1: 14–19 years, site 2: 18–25 years free of ice). The comparison of sequencing data retrieved from in‐growth mesh‐bag (MB) samples compared to soil samples was carried out for winter samples in site 2. Both winter soil samples (with snow cover and 2 weeks after snow‐melt) have a highly similar pattern compared to the MB sample from the same site. Black bars indicate the snow‐free period, white bars the snow‐covered period. [Color figure can be viewed at http://wileyonlinelibrary.com]

The comparison of soil samples retrieved from under snow‐cover, to soil samples sampled 2 weeks after snowmelt showed no significant differences. Fungal communities from the same plot clustered close to each other, irrespective of sampling date (Fig. 6).

### *Fungal community composition shifts radically between the earliest SSD*

On the community level (beta‐diversity), different SSD formed distinct clusters. Differences in community composition are highly significant, as shown by both ANOSIM and PERMANOVA (*p* < 0.001, Supporting Information Table S4), providing proof for a distinct succession of fungal communities. The earliest SSD (site 0) is most distinctive regarding species composition compared to sites 1 and 2 (Figs. 5 and 6). The majority of OTUs did not appear in site 0 at all, or at least, not in a regular pattern. Rather, many OTUs started to first appear in site 1 and stayed present along the successional gradient (e.g., most *Tetracladium* OTUs, some Helotiales, *Dioszegia*).

Ascomycota, Basidiomycota and Mortierellomycota were found in all SSDs, with Ascomycota clearly dominating in relative abundance. However, the variability of abundances in plots of the same group (SSD and season), especially for Ascomycota was high, for example, spanning in a range of 22%–98% of reads in site 1 in summer. in site 2 during winter, however, the Ascomycota reads were generally dominating, ranging from 74% to 95% of total reads per plot and within‐group variability for the phylum was low. Similarly, Basidiomycota tended to show smaller within‐group variability in site 2 during winter, however, concentrating their relative abundance in the lower abundance area (2%–12%). The relative abundance of Basidiomycota decreased in older SSDs during winter. Chytridiomycota were almost absent in site 0 but found in high relative abundance in single plots of sites 1 and 2. Another group of zoosporic fungi, the Monoblepharidomycota, was almost exclusively found in the earliest SSD, with a slightly higher relative abundance in summer (Supporting Information Fig. S1).

### *Seasonal community shifts are typical, and most pronounced in barren, recently deglaciated sites*

Fungal communities of all SSDs also differed significantly between summer and winter (ANOSIM and PERMANOVA *p* < 0.05; Supporting Information Table S4). Ordination plots of fungal communities showed distinct clusters for the individual successional stages and seasons, apart from individual sample MB‐S2 and the outlier samples of plot 23 (Fig. 5). However, the separation by season became less prominent in the latest SSD (site 2), where clusters started to intermix. The odd placement of plot 23 samples can be explained by the location of this plot directly in line of a melting water stream. The deviation of sample MB‐S2 could not be explained, but it highlights local heterogeneities as typical for alpine sites.

**Figure 7:**
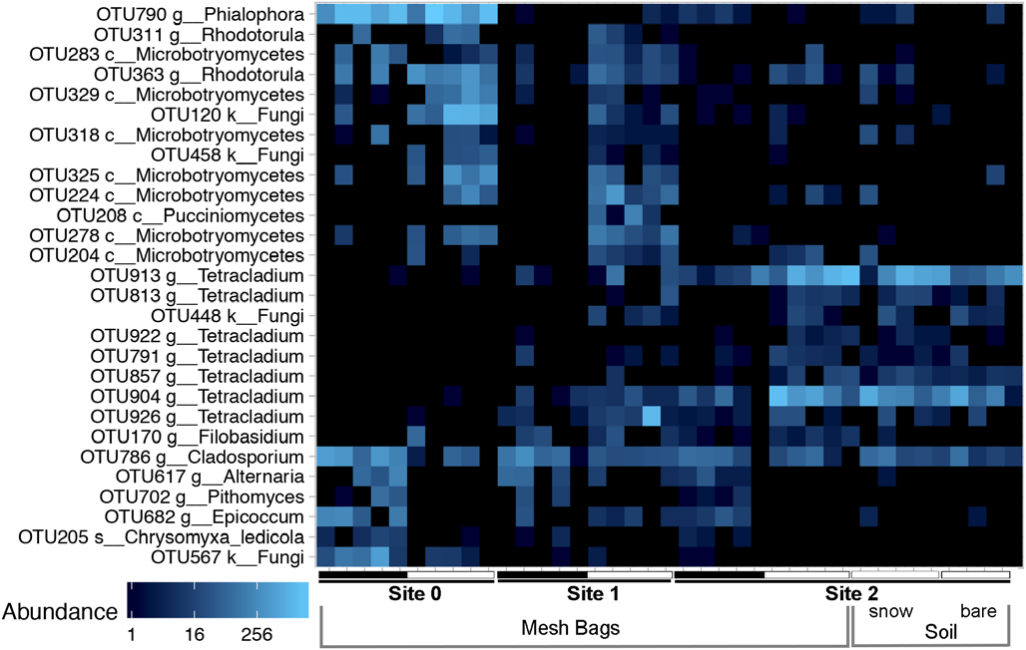
Heat map of fungal OTUs detected in MBs showing significant differential abundances (raw *p*‐values, without multiple testing correction) in at least one of the three stages of soil development (site 0: 0–3 years, site 1: 14–19 years, site 2: 18–25 years free of ice). Winter fungal communities are most similar during the first two stages of soil development. The comparison of sequencing data retrieved from in‐growth mesh‐bags (MB) compared to soil samples was carried out for winter samples in site 2. Data retrieved from soil samples are very similar to data retrieved from MBs. Black bars indicate the snow‐free period, white bars the snow‐covered period. [Color figure can be viewed at http://wileyonlinelibrary.com]

Some fungal taxa, especially of the class Microbotryomycetes (including *Rhodotorula*) showed a high abundance during the winter season in both sites 0 and 1 but were rare in the same plots during summer. Differential abundance testing (Welch's *t*‐test) resulted in no significant differences for any OTUs when using FDR‐correction. However, when omitting multiple‐testing corrections, the graphic display of significant OTUs (raw *p*‐value < 0.05) produced a strong visual pattern of contrasting seasonal abundances for several OTUs (Fig. 7). In total, 28 OTUs displayed a seasonal preference in at least one of the three SSDs. For OTUs of the class Microbotryomycetes, the pattern is present over two consecutive successional stages (sites 0 and 1), reinforcing the hypothesis that these OTUs prefer one of the two contrasting seasons. This pattern is only indicative and not statistically significant. Especially in habitats with high heterogeneity, multiple‐testing corrections might often be too stringent if sampling efficiency not high enough.

### *Amplicon sequencing detects a higher diversity, but rare fungal isolates are not always detected*

Amplicon sequencing detected a higher fungal diversity than the cultivation approach (220 OTUs vs. 91 cOTUs). Whole taxonomic groups were not detectable using cultivation techniques (e.g., Chytridio‐, Rozello‐, Monoblepharido‐ and Glomeromycota). However, 24 cOTUs did not have a close match in the amplicon sequencing data set (BLAST), which means that they were not detected. Most of these cOTUs were rare: 9 cOTUs were isolated only once and 14 cOTUs were isolated twice (Supporting Information Table S5).

## Discussion

### *Soil fungal communities closest to the glacier tongue are diverse and unique and rather resembling microbial communities of cryoconite than of soil*

We used amplicon sequencing and isolation of fungi from in‐growth MBs, to identify active fungi in a rather marginally addressed transition phase from cryosphere to early soil development (0–25 years).

As soon as 2 years after melting of the ice sheet, specific community‐style patterns of fungal mycobiota were detected on barren ground in the glacier forefield. Ascomycetes (*Phialophora, Sarocladium, Mortierella*) and psychrophilic basidiomycete yeasts preferentially colonized soil closest to the glacier. The latter were also reported to be highly abundant in other alpine glacier forefields (Rime *et al.,* 2015), indicating an important role of these fungi as primary colonizers of mineral soil in the glacier forefield. Of all OTUs detected via amplicon sequencing, 35% were exclusively found in site 0. These early colonizers confirm a clear successional trend in the earliest SSD, because most of them disappeared in later SSDs, especially site 2. They are especially interesting, as they represent the transition from life adapted to ice to life adapted to soil. Soil fungal communities closest to the glacier tongue are unique and rather resembling microbial communities of cryoconite than of soil (Edwards *et al.,* 2013). This implies that glacial environments might be important reservoirs of fungal diversity. Edwards *et al*. (2013) also described a potential aeroaquatic‐endophyte cycle for cryoconite‐associated Ingoldian fungi. We detected a high diversity of the genus *Tetracladium*, a typical representative of Ingoldian fungi. Cryoconite as inoculum explains very convincingly the high abundance of these endophytes in soil, as soon as the first pioneer plants occur, and further strengthens our hypothesis for dispersal between cryoconite and early soil.

The earliest hyphomycete communities are usually characterized by typical representative species of Helotiales (Rime *et al.,* 2015). We detected one of them, namely a *Phialophora* species (OTU 790) almost exclusively in soil close to the glacier tongue (site 0), with high abundance throughout the year. The related ITS2 sequence was identical (100% identity, 100% coverage) to environmental sequences from arctic and alpine regions (Midre Lovenbreen Glacier Norway JN113039, Qinghai‐Tibet plateau JX001631). The corresponding UNITE species hypothesis (SH013873.07FU) is typical for cold regions and was reported from Antarctica, the North American Arctic, Svalbard and Tibet. Besides being considered as a saprobe, it was also reported as endophyte of *Chorisodontium aciphyllum*, a moss frequently found along the Antarctic coast in the Drake Passage region (Zhang *et al.,* 2013). Morphological characteristics of the related isolates (cOTU97) indicate that this could possibly be a new species of the recently described genus *Psychrophila* (Wang *et al.,* 2015).

Drastic changes in fungal species composition coincide with the occurrence of first pioneer plants and the build‐up of SOM. An increased nutrient availability due to SOM build‐up has been shown to drastically influence yeast diversity in alpine glaciers (Turchetti *et al.,* 2013). On the Rotmoos glacier forefront, a 4‐year period of ecosystem development (corresponding to our site 0) is too short to enable plant species to colonize bare soil. After about 10 years of exposure, soil is scattered with single‐vascular plants (e.g., *Saxifraga aizoides*), and a high percentage of fungal species now appear for the first time on site, remaining present as soil development proceeds. Many of these fungal taxa are known for their various associations with plants, for example, as endophytes or pathogens (Helotiales, *Botrytis* and *Neonetrica*).

### *Fungal communities follow a distinct successional pattern*

The observed increase in fungal species richness and diversity with successional age appears to be typical for glacier foreland successional sites: Soil fungal community development is reaching a mid‐successional diversity maximum at about 40 years of soil development (Dong *et al.,* 2016). Three recent successional studies carried out in glacier forefields from Tierra del Fuego (Chile) (Fernandez‐Martinez *et al.,* 2017), the Altai mountains (Russia) (Gatti *et al.,* 2018) and Tibet (China) (Tian *et al.,* 2017) also included the earliest sites in close vicinity to the glacier tongue. These studies share our observation that earliest fungal communities from barren ground closest to the glacier terminus are clearly segregated from the rest of the chronosequence. These abrupt changes in community composition at early SSD are also prevalent for other pioneer organisms like bacteria and algae.

### *Seasonality is especially prominent in the earliest SSDs*

The fact that fungi are actively growing in soil during winter has been widely neglected for a long time, although early reported by Gams (1960). The rediscovery of fungal communities which are physiologically active under snow‐cover (Schadt *et al.,* 2003) caused a renewal of interest in the scientific community, leading to the discovery of many unknown fungal species or even lineages. On this basis, an earlier study on fungal seasonal dynamics was carried out at a late successional site (~150 years of soil development) of the Rotmoosferner glacier forefront. This study demonstrated that fungal biomass can increase more than 10‐fold underneath the snow cover (Kuhnert *et al.,* 2012). We also observed a change in soil fungal community composition. Seasonal shifts in soil fungal species composition are also known for other well‐developed soils (Voříšková and Baldrian, 2013; Voříšková *et al.,* 2014; Žifčáková *et al.,* 2016).

In the present study, we focused on seasonal dynamics in the earliest SSD and detected clear seasonal shifts in fungal community composition between the short summer and the long winter period. However, seasonality became less prominent in the latest SSD. Several fungal taxa with seasonal preferences were detected: OTUs with higher abundance in winter (especially in early SSDs 0, 1) were predominantly found in the class of Microbotryomycetes. Taxonomic resolution at the genus level was available only for OTUs belong to the genus *Rhodotorula* (psychrotolerant yeasts). Strains of Microbotryomycetes (cOTUs 62 and 64) were also frequently isolated from winter MBs buried in these sites. They were preliminarily identified at the genus level and are probably representing new psychrophilic yeast species of *Cryptococcus*, *Rhodotorula* and *Mrakia*. Studies on cultivable yeast communities from other Alpine glaciers (Glacier du Geant, France, and Miage Glacier, Italy) (Turchetti *et al.,* 2013) confirm that distinct psychrotolerant basidiomycete yeasts are representing a stable core of the sub‐glacial yeast communities; these communities are not only typical for glaciers (Butinar *et al.,* 2007; Branda *et al.,* 2010), cryoconite (Margesin *et al.,* 2002; Singh and Singh, 2012), but also for glacial melting water (De García *et al.,* 2007) and arctic coastal environments (Butinar *et al.,* 2011). This implies that cryoconite and melting water must be considered as a possible inoculum for the native soil close to the glacier tongue.

Ascomycota growing as hyphomycetes were found to dominate winter communities in site 2 (18–25 years of soil development), where they made up two‐third of the mycobiota over all plots; especially, various OTUs of the genus *Tetracladium* and other non‐closer defined OTUs within the same order Helotiales were responsible for these high abundance values.

The detection of a biotrophic plant pathogenic fungus belonging to Pucciniomycetes (OTU 208) in the intermediate SSD (site 1) during winter only was surprising. This could suggest a saprobial lifestyle during winter, when the host is not active or alive, and a switch back to biotrophic lifestyle as soon as the host is physiologically active again during the vegetation period. Due to the presence of this OTU in 4 of 5 plots a contamination is highly unlikely.

Furthermore, we also detected *Chrysomyxa* sp. (OTU 205), another biotrophic rust fungus in low relative abundances (<1%) in all SSDs, but especially in barren ground (site 0: 4 plots in summer, 1 plot in winter). Due to the obvious absence of vascular plants, we assume an alternative lifestyle (e.g., saprobial yeast) or unknown intermediate host (e.g., algae). This indicates that the teliospores (the thick‐walled resting spores of rust fungi) produce basidia and basidiospores in snow‐covered soil. Basidiospores of rusts bud and persist as yeasts (Aime *et al.,* 2014), and they have been detected in soil habitats (Stefani *et al.,* 2010). Habitats of host generalist and specialist fungi (both pathogens and mutualists) can exceed those of their host species (Merges *et al*. 2018). However, the presence of a host plant has indeed a positive effect on the abundance of host‐specialized pathogenic fungi.

However, we also found several OTUs displaying higher relative abundances in summer than winter. *Alternaria, Epicoccum* and *Pithomyces* were found to be more active during the vegetation period. In addition, we detected a species of *Cladosporium* (OTU 786), which was active in both seasons over all SSDs, but whose abundance was generally higher in summer than in winter (Fig. 7). *Cladosporium* species have multiple relationships with plants (endophytes, epiphytes, pathogens) (Bensch 2012), explaining higher abundances during the vegetation period.

Several patterns of seasonal preference were visible over multiple successional stages, but not detected when applying multiple‐testing correction (FDR). This indicates that FDR was too stringent in this case and stresses how the visualization of significant results without multiple‐testing correction might prevent missing relevant biological information.

### *In‐growth MBs allow for a meaningful detection and isolation of winter‐active fungi, although having some drawbacks*

In‐growth MBs are generally used to capture biomass of hyphae‐forming fungi, which were actively growing during the incubation period. The biomass colonizing or growing through the sterile substrate (quartz‐sand) is then used for isolation or DNA‐based identification of fungi. The use of MBs instead of soil samples aims for preventing detection/isolation of inactive (spores, sclerotia) or dead fungal material (Wallander *et al.,* 2001). In addition, fungal hyphae can be visualized and quantified from MB content (Kuhnert *et al.,* 2012).

Although the MB approach might be suitable to detect and quantify in‐growth of hyphae‐forming fungi, we did notice problems occurring with non‐hyphal, single‐celled organisms. Yeasts or spores can be washed into MBs with a mesh size of 53 μm and revive upon isolation/cultivation procedure. A smaller mesh size should be used to prevent that in the future.

### *The in‐growth MB approach is an interesting alternative to soil sampling, when trying to assess fungal organisms growing during defined periods*

Based on amplicon sequencing of winter MBs and the surrounding soil, we could not detect any significant differences in fungal community composition (ANOSIM, PERMANOVA). This was surprising, because we expected a higher diversity in soil samples due to co‐amplification of inactive fungal structures. We hypothesise that fungal inoculum is either generally introduced into this environment in very small amounts only, or that it is quickly removed from the system by high turnover rates, as introduced fungal propagules might easily get washed out, blown away or consumed by soil invertebrates. This means that fungal spore banks have not been established yet in these SSD. Based on differential abundance test of soil versus MBs samples, only two OTUs were exclusively found in soil and one exclusively in MBs: A *Cadophora*‐/*Phialophora*‐like fungus (OTU875) appeared to be typical for summer MB samples but was absent in most (4/5) winter MBs. Still, this fungus appeared to be frequent in winter soil samples (4/5), but only in those retrieved from under the snow cover. *Cadophora*‐/*Phialophora*‐like fungi are typical dark septate endophytes, which are frequently occurring in the roots of arctic/alpine pioneer plants on a global scale (Jummponen & Trappe 1998; Hewitt *et al*. 2017). During summer, (extra‐radical) mycelia are foraging through the ground, explaining why they are frequently detected in MBs. During winter, roots harbouring these fungi are degraded and spores are liberated in the soil, where they were detected. They were not growing and therefore not detected as in winter MBs. In this case, MBs might have worked as intended by excluding inactive fungal propagules. Low abundances in combination with high turnover rates or washout could explain why these were not detected in soil shortly after snow melt. *Murispora* (OTU725) **a plant‐associated, dothideomycete is known for its large, muriform ascospores. This OTU was detected in summer MBs from middle and late SSDs and in snow‐covered soil samples. Arbuscular mycorrhizal fungi (Glomeromycota, OTU364) were only detected in sites with plant cover (site 2). At this site, they were detected in summer MBs as well as in winter soil samples, however, not in winter MBs. The spores of arbuscular mycorrhizal fungi are typically very large (50–200 μm) and therefore too big to enter MBs. Still, they are present in the surrounding soil as inactive states and could therefore easily be detected in winter soil samples. This indicates that the concept of assessing only actively growing fungi by using in‐growth MBs is working for Glomeromycota with a spore size above mesh size.

Summing up, the MB approach can be regarded as an interesting alternative to soil sampling, when trying to assess active fungal organisms during certain seasons. However, yeasts and small fungal spores pose problems to this method. Due to the large mesh‐size used (53 μm), most detected OTUs could also have been washed into MBs. We found no differences in the retrieved fungal communities with the two sampling strategies (MBs vs. soil), but we compared only winter MBs with the surrounding soil, and this is a habitat with extremely low SOM content. Results might be different in other environments. Nevertheless, we regard the MB approach as very valuable, especially for the isolation and cultivation of fungi. It might also be appropriate for studying the activity of specific fungal groups (e.g., Glomeromycota).

### *Species turnover takes more than 2 weeks after snow‐melt, showing that there are no short‐term effects of snow melt on soil fungal communities*

The comparison of fungal communities derived from snow‐covered versus 2‐weeks snow‐free soil samples in SSD 2 did not show any significant differences between these two time points. Thus, it can be assumed that changes in community composition take several weeks. Factors affecting the rate of species turnover are still obscure, but there is obviously a lag‐phase between seasons. Nutrient availability or the slow development of trophic interactions after snowmelt could be one important factor. Snowmelt itself might intensify the nutrient scarcity due to washout by melting water. Still, it has to be considered that this comparison was carried out only for the most developed SSD—site 2. In this stage, the seasonal shift of soil fungal communities was not as prominent as in less developed SSDs. Site 2 already has a mosaic‐like plant cover with a substantial amount of organic matter, able to hold water and nutrients on site. Such organic layers were only present as isolated colonies in site 1 and were completely lacking in site 0. The investigation of this short‐term effect was included in our study due to an unexpected event in the field study: An avalanche had knocked down the snow poles, which made the localization of plots from site 1 under the snow cover impossible. Winter samples could therefore be retrieved immediately after snowmelt, only. To estimate the effect of this late retrieval, we used site 2 as the nearest proxy for a comparison of short‐term effects following snowmelt. The results clearly show that 2 weeks of being snow‐free does not significantly influence fungal community composition. This was important for validation of the winter samples of site 1.

### *Cultivation of winter‐active fungi is highly biased but has immense importance and potential*

Results of cultivation‐based and cultivation‐free methods were widely in accordance, at least when considering abundant fungal taxa. However, there is a restriction to fungal groups, which can be isolated with the methods applied, and the cultivation approach is much more tedious and time consuming than the DNA‐based approach. Therefore, it is not surprising that fungal diversity studies based on targeted environmental sequencing combined with cultivation are comparatively rare (e.g., Jebaraj *et al.,* 2010).

As expected, the proportion of psychrophilic fungi (no growth at 25°C) among isolates (cOTUs) was generally high, considering the soil temperatures range between 0°C and 10°C throughout the year in the glacier forefield. Several cultivation approaches were tested to find the best, but most effective way of cultivation. The results of cultivation‐based methods were strongly depending on the applied method of isolation (direct vs. dilution plating). Thus, the combination of both methods might provide a better picture of the fungal diversity. The enormous workload of cultivation and identification of isolate does not allow for extensive replicates, and thus, a statistical evaluation of the results is not possible. However, it enables an estimation of the diversity of the most abundant fungi in a habitat. More importantly, it allows for a thorough study of the obtained isolates. This is especially important considering the high degree of unknown biology of the identified fungal taxa. It is very likely that most of our isolates are saprobial, but representatives for many other functional groups were also isolated: for example, potentially mycorrhizal (*Russuales* sp.1, *Sebacinaceae sp*.), plant endophytes (*Tetracladium* spp.), plant pathogens (*Taphrinaceae sp*., *Heterobasidion* sp.) and entomopathogenic fungi (*Lecanicillium spp*., Hypocreales sp.). Fungal isolates do not only offer the possibility to study, test and describe new fungal taxa. They also offer the possibility to test strains for a variety of applications of psychrophilic fungi, for example, for special cold‐stable enzymes. The number of cOTUs obtained via isolation was highest in the youngest SSD, which is in contrast to the NGS data, where fungal richness increased along the successional gradient (cf. rarefaction plots). This could either be explained by the lack of ‘ecological’ replicates for each SSD (only one plot per season and SSD tested). However, we rather prefer a second hypothesis, namely that this bias was caused by the strong selectivity of cultivation methods in contrast to cultivation‐free methods. Along the chronosequence, we find more and more fungal groups strictly associated to plants. Many of these plant‐associated fungi rely on interactions with their hosts for growth, for example, biotrophic parasites or mycorrhizal fungi, and can therefore not be cultivated. Thus, species diversity of cultivable fungi decreases with increased proportion of plant‐associated fungi. When assessing fungal diversity, amplicon NGS is without question the more sensitive method than cultivation‐based approaches. However, more than a quarter of the cOTUs (27%) did not have a matching BLAST reference in the amplicon data set (Supporting Information Table S5). The number of cOTUs without match could be reduced when using the non‐filtered dataset as reference, although. Nevertheless, 17 cOTUs could still not be detected by NGS sequencing. Their absence could be explained by their low abundance (30% of all isolates were retrieved only once), and the stringent quality control measures in the NGS data analysis. Furthermore, there are several technical issues related to NGS generally, which are known to alter the recorded biodiversity. These include biases regarding DNA extraction, PCR primers, variation in rDNA copy numbers per genome, number of nuclei per ‘individual’, and so forth; the use of abundance of sequence reads as a proxy for abundance of individuals is working fine for the most abundant taxa but is far from perfect (Amend *et al*., 2010, Nguyen *et al*., 2015).

### *Conclusion*

Global warming accelerates glacier retreat and causes a reduction of snow‐covered landscapes on a global scale. Snow cover is an excellent thermal insulator, keeping the ground temperature warmer than the air temperature during cold seasons. This insulation effect enables and promotes the growth of the winter‐active soil microbiota. A pronounced seasonal species turnover between snow covered soil and soil with plant cover has already been reported for different habitats (e.g., forests, agricultural soil, tundra soil). However, this is the first study showing that fungal diversity is not only comparatively high in barren ground closest to the glacier tongue, but it is also subject to distinct seasonal species turnover. Environmental studies are usually based on sampling during the vegetation period only. However, by doing so, the generated data reveal only one side of the coin, and a high proportion of the real microbial diversity and function remains overlooked.

Fungal communities of recently deglaciated soil are significantly different from communities of later SSD and are rather similar to fungal communities typical for cryoconite or ice. This indicates different inoculum sources for the earliest ice‐related barren ground and for later plant‐covered soil. The highly dynamic ice surfaces of a glacier are characterized by continuous melting and freezing. It is therefore very likely that the earliest ice‐free barren ground obtains its fungal inoculum from cryoconite via wind‐borne propagules or via fungal structures transported by melting water.

## Experimental procedures

### *Sampling site description*

The study site is located in the forefield of the Rotmoos glacier, in the Central Austrian Alps (Ötztal, 46°50′ N, 11°03′ E) at 2280–2450 m above sea level. Since 1858, the Rotmoos glacier has retreated for about 2 km, and due to its low slope and wide profile, the forefield is well suited for studies of primary succession (Erschbamer and Nagl, 2010). Towards the glacier tongue, all soils are influenced by the basement rocks of the Ötztal‐Stubai complex and by the Schneeberg complex (part of the Austrian nappe stack system), which are a remnant of a strongly carbonate‐influenced sediment layer (Krainer, 2010; Tropper *et al.,* 2012).

Three areas with distinct SSD (sites 0, 1, 2) were chosen close to the glacier tongue for comparative studies of active soil mycobiota in the earliest stages of soil formation. The three different sites have been free from glacial ice for 0–3 years (site 0), 9–14 years (site 1) and 18–25 years (site 2) respectively. The SSD reach from non‐vegetated, barren ground (site 0), over barren ground colonized by individual pioneer plants (site 1), to soil with a partial, mosaic‐like vegetation cover (site 2) (Fig. 1). In each site, eight representative subplots of 1 m^2^ were chosen as random replicates for each SSD. Soils are usually covered with snow from mid‐October to late May (Kaufmann, 2001). Soil temperatures range from −0.6 to −0.1°C during the snow‐covered period and from 0 to 22°C during the vegetation period (Kuhnert *et al.,* 2012).

### *Sampling strategy*

In each plot (8 per SSD), eight in‐growth MBs were incubated for a season of prolonged snow‐cover (referred to as ‘winter MBs’) or the vegetation period (‘summer MBs’) respectively to assess active fungal communities in two contrasting seasons. Active hyphae‐forming fungi move through the soil by growth. Occasionally, these hyphae will pass through the randomly distributed MBs, leaving behind biomass on the filling (acid‐washed quartz sand). The biomass can then be used either for the isolation of cultivatable fungi or for their detection via DNA‐based methods (Wallander *et al.,* 2001). The applied MBs were comparatively small (1 g quartz‐sand), facilitating nutrient diffusion, as well as easy passage of fungi. Summer MBs were buried in soil in mid‐July (11^th^ and 16^th^) 2015, incubated for 2 months during the vegetation period and retrieved on September 18, 2015. Winter MBs were buried in soil on September 18, 2015, and incubated during winter. Plots were marked with 2 m long aluminium rods (serving as snow poles) to enable localization underneath the snow cover. Winter MBs were retrieved from soil while still covered by snow in June and July 2016, with the exception of plot W2 (site 0) and all plots of site 1, which were sampled about 2 weeks after snow melt. An avalanche had knocked down the aluminium rods of site 1 and made localization of plots under the snow sheet unfeasible. Upon retrieval, MBs from each plot were placed into separate plastic bags to avoid cross‐contamination and stored on ice during transportation and sample processing. Soil samples for physicochemical analyses were retrieved at the beginning of the vegetation period in June 2015. in site 2 (18–25 years ice free), additional soil samples for DNA‐based analyses were retrieved from both, snow‐covered soil and 2 weeks after snow melt in 2016, to check for differences between MB and soil samples, as well as for short‐term changes in fungal communities upon snow melt.

### *Soil physicochemical analyses*

From each plot, 5–8 small spades of soil (ca. 30 g in total) were taken randomly from 1–5 cm depth. Samples from each plot were mixed, sieved to 2 mm and stored at −20°C. During analytical procedures, soil was stored at 4°C in polyethylene bags. Soil pH was measured potentiometrically in 0.01 M CaCl_2_. Soil dry weight was determined by drying samples at 105°C. The determination of organic matter by the loss‐on‐ignition method and the semi‐quantitatively estimation of carbonate (CaCO_3_) were carried out as described by Schlichting *et al*. (1995). Ammonium (NH_4_), nitrate (NO_3_), plant‐available and total phosphate (PO_4_) were measured as described by Schinner et al. (1996). Soil physicochemical data were analysed in STATISTICA (ver. 9.1 Statsoft Inc.). Normal distribution of variables was checked via QQ‐plots. Depending on distribution, either a parametric ANOVA and Tukey HSD test or non‐parametric Kruskal‐Wallis tests were used to examine differences of variables between soil development stages (sites 0, 1, 2). Furthermore, spearman correlations were calculated between all soil parameters over all sites.

### *Cultivation of soil fungi from in‐growth MBs*

Fungi were isolated from MBs by direct‐plating (Czaban and Wróblewska 2006) and dilution plating (Turchetti *et al.,* 2013). For each site and season, a single plot was chosen, of which three MBs were processed. For direct plating, 30 quartz‐sand grains were used per MB and medium. For dilution plating, 1 g of quartz‐sand particles was suspended 1:10 (w/v) in 0.1% tetrasodium pyrophosphate and shaken overhead for 30 min. Serial dilutions (10^−1^ to 10^−3^) were prepared, and 100 μl were plated on potato dextrose agar (PDA, Carl Roth, Germany) and synthetic nutrient‐poor agar (SNA), with three replicates for each medium. To suppress bacterial growth, media were supplemented with antibiotics (100 mg L^−1^ streptomycin, 50 mg L^−1^ tetracycline). Plates were incubated at 4°C and 10°C in parallel for >4 weeks and checked for growth regularly. Pure cultures were transferred to PDA without antibiotics and further incubated at both the isolation temperature and at 25°C to allow for a standardized morphological investigation and determination of psychrophilic growth. Morphological identification of isolates was based on general literature for soil fungi (Domsch *et al*. 2008) and monographs on the respective genera (Linnemann 1941). Moreover, sequencing of rDNA ITS barcoding region was performed via direct colony PCR as described by (Walch *et al*. 2016) using the primers ITS1 and ITS4 (White *et al.,* 1990). If direct colony PCR failed, DNA was extracted from pure cultures using the E.Z.N.A. HP Fungal DNA Kit (Omega Biotek) before PCR amplification. Sequencing of PCR products was carried out at MicroSynth AG (Switzerland). Quality‐checked sequences were assembled into ‘cultivated operational taxonomic units’ (cOTUs, term to discern from NGS‐generated OTUs) at 99% and 97% sequence similarity using Sequencher (ver. 5.4.6; Gene Codes Corp.). Taxonomy was assigned by closest hits after manual BLAST runs against International Nucleotide Sequence Database** Collaboration (INSDC; http://www.insdc.org/ (Karsch‐Mizrachi *et al.,* 2018) and UNITE (Kõljalg *et al.,* 2013). A representative sequence of each 99% OTU was submitted to GenBank (Supporting Information Table S3). Pure cultures are stored in the culture collection at the University of Innsbruck, Institute of Microbiology (Supporting Information Table S3).

The presence/absence data of isolated fungi were used to check if patterns of fungal activity derived from the cultivation approach are in concordance with amplicon data. Furthermore, the ITS2 region of each 97% OTU was extracted using ITSx and blasted locally against reference sequences of the filtered NGS data set to check if cultivation can detect OTUs that might go unnoticed by the NGS approach.

### *DNA extraction and amplicon sequencing*

To reduce the effect of small‐scale habitat heterogeneity, the content of three MBs per plot were pooled and used for DNA extraction. MBs were opened with a flame‐sterilized scalpel, and the content was poured into a sterile petri dish. Five ml of quartz sand (roughly content of 3 MBs) were filled into a 5 ml Eppendorf tube, and 3 ml extraction buffer were added. Tubes were sealed with Parafilm® and put on a vortex mixer for 5 min at 3000 rpm. The addition of an extra bead‐beating matrix was omitted as the MB samples consist of sharp grains (silicate), which were expected to shear attached biomass. The lysate was incubated at 65°C for 60 min and regularly mixed by inverting tubes every 10 min. The liquid lysate (~ 2 ml) was withdrawn with a sterile plastic syringe (Braun Injekt™) and needle (Braun Sterican™ 20 G × 1.5″). Keeping sample amount high and buffer volume low was necessary to obtain a sufficient concentration of DNA. The lysate (1.2 ml) was aliquoted into two 1.5 ml tubes, and extraction was continued with the E.Z.N.A. HP Fungal DNA Kit (Omega Bio‐tek). The protocol was modified by including a column equilibration with 3 M NaOH (optional, Product manual November 2015) and the withdrawal of 400 μl of aqueous phase (instead of 300 μl) following chloroform‐isoamyl alcohol extraction to increase yield. Volumes of the CXD buffer and ethanol were adjusted accordingly. Tubes were incubated 5 min at 65°C upon addition of elution buffer to increase DNA yield. Extraction success was checked by DNA concentration measurement using Picogreen® and by test PCR reactions targeting the ITS2 region. Amplification success was checked via gel electrophoresis. Based on the results of test PCRs (criterion: strong product in both summer and winter MB samples), five plots of each SSD (sites 0, 1, 2) were selected for fungal community profiling using amplicon metagenomics.

For soil DNA, 250 mg of sieved soil were used for DNA extraction using the E.Z.N.A. Soil DNA kit (Omega Bio‐tek). Soil samples were included for comparison with MB data. One SSD and one season (site 2, winter) were used for this comparison, leading to 40 NGS‐analysed samples in total.

A two‐step PCR protocol was used to generate sequencing libraries targeting the rDNA ITS2 region. The first reaction was carried out in a volume of 25 μl, with 5 μl template, 1× KAPA HiFi buffer, 0.2 mg ml^−1^ bovine serum albumine, 0.3 mM of each dNTP, 0.3 μM of each forward and reverse primer and 1 unit of a high‐fidelity polymerase (KAPA HiFi HotStart, Kapa Biosystems). Primers consisted of a locus‐specific sequence (ITS3, ITS4 (White *et al.,* 1990) and the Illumina adapter). For the forward primer (ITS3), five random bases (N) were included in between the primer regions to increase sequence diversity and cluster template registration by the Illumina system. This increases overall sequencing quality of low diversity libraries runs like any amplicon‐based library (Anonymous, 2018). After initial denaturation at 95°C for 4 min, PCR was performed with 25 cycles of 98°C for 20 s, 55°C for 30 s and 72°C for 30 s, with a final elongation of 5 min at 72°C. PCR was performed in triplicates for each sample to reduce PCR‐related biases and increase sensitivity (Schmidt *et al.,* 2008). Triplicates were pooled and cleaned up using the GeneElute™ PCR Clean‐Up Kit (Sigma‐Aldrich). Purified PCR products were sent to Microsynth AG (Switzerland) for second step PCR. The number of cycles in the second step PCR was raised from 15 to 20 to increase yield. Libraries were pooled in an equimolar manner, and paired‐end sequencing (2 × 250 bp) was conducted on an Illumina MiSeq platform. Sequencing results were provided as de‐multiplexed fastq files.

### *Bioinformatics and statistical analysis of amplicon data*

Raw sequence reads (*n* = 1,874,918, forward and reverse) were processed with the pipeline PIPITS (ver. 1.5.0, Gweon *et al*.) on a standard desktop PC with 8 GB RAM running Ubuntu 16.04. The fungal ITS2 region was extracted using ITSx to increase resolution in downstream sequence clustering and remove reads of non‐fungal origin (Bengtsson‐Palme *et al.,* 2013). Short (<100 bp) and unique sequences were removed before OTU finding, as these are likely to have emerged as sequencing errors (Edgar, 2013). Sequences were clustered to OTUs at 97% sequence identity. Chimera detection was run with UCHIME (Edgar *et al.,* 2011) based on the UNITE UCHIME reference dataset (01.01.2016). Taxonomy was assigned using the RDP classifier and the latest trained UNITE fungal ITS reference dataset (UNITE 7.2; 28.06.2017) as implemented in PIPITS. Sequence data were deposited in the European Nucleotide Archive (ENA) under the project accession PRJEB30879. The biom file produced by PIPITS was combined with sample metadata using biom and analysed in R (3.3.2) (Supporting Information Table S5). Non‐fungal lineages overseen by ITSx (*n* = 20, mostly Chromista) were removed from the dataset. Rarefaction plots were drawn to check whether sequencing depth sufficiently covered fungal diversity (Supporting Information Fig. S2). Based on these results, all samples were rarefied (subsampled with replacement) to 4020 reads per sample (size of second smallest sample) to mitigate the effect of uneven sample sizes on downstream analyses (Weiss *et al*. 2017), while retaining as much sequence diversity as possible. Furthermore, only OTUs with an abundance of at least 3 reads in a minimum of 3 samples (note: total number of replicates per SSD and season is 5) were retained to remove low abundance OTUs that might represent contaminations. For the filtered dataset, manual BLASTn searches were run against the International Nucleotide Sequence Database Collaboration (INSDC) with exclusion of uncultured and environmental sample sequences. Taxonomy was assigned up to genus level, when a reliable match was obtained within 97% sequence identity and > 90% coverage. Hits were considered reliable when multiple references displayed the same or equivalent results. The results provided by the UNITE and NCBI databases were checked for concordance in the assigned taxonomy. If NCBI provided a reliable higher taxonomic resolution, the taxonomy was adjusted in the data. Fungal richness and diversity was investigated based on observed OTUs and the Shannon‐diversity index. Differences between diversity indices were statistically evaluated using ANOVA and post hoc tests (Tukey‐HSD). Data requirements for parametric tests have been confirmed beforehand (Shapiro–Wilk test). Dissimilarities between samples were displayed by an ordination‐based heat map (Rajaram and Oono, 2010) and by non‐metric multidimensional scaling (NMDS) plots, based on a Bray–Curtis dissimilarity matrix as calculated from the rarefied, filtered and square root transformed OTU abundance data. Venn diagrams were used to show shared species between SSDs for periods of snow‐cover (W) and the vegetation period (S) respectively (Supporting Information Fig. S3). Differences among a‐priori defined groups were tested by analysis of similarity (ANOSIM) and permutational non‐parametric MANOVA (PERMANOVA). Homogeneity of dispersion among groups was confirmed (Legendre and Anderson, 1999) using the function betadisper in the R package vegan. This is necessary as significant differences detected by PERMANOVA may arise due to differences in dispersions between compared groups (Anderson, 2006). To test for the main working hypotheses the following subsets were analysed individually using ANOSIM and PERMANOVA: (i) >MB samples< to investigate the effect of season and SSD on fungal community composition. (ii) >Soil samples< to compare fungal community composition in soil samples with or without snow‐cover (short‐term effects). (iii) >Winter samples from site 2 taken from beneath snow‐cover< to compare fungal communities retrieved from MBs versus soil samples (methodological comparison). For the MB samples, unequal variances *t*‐tests (‘Welch's t‐test’) were conducted for each SSD separately to identify OTUs with a significant difference in their mean abundance between seasons. A correction for false discovery rate (FDR; Benjamini & Hochberg 1995) was included in multiple testing. OTUs, which displayed a seasonal pattern in at least one of the three SSD where visualized in a heat map (even if the FDR‐adjusted *p*‐values were >0.05, if raw *p*‐values were <0.05).

## Conflict of interest

The authors have no conflict of interest to declare.

## Supporting information


**Appendix S1**: Supporting Information

**Fig. S 1** Relative seasonal abundances of fungal phyla occurring in earliest stages of soil development ranging from 0 to 25 years (site 0, site 1 and site 2) based on amplicon sequencing data. Ascomycota dominate during early soil development. But especially during the first two stages of soil development, relative abundances of Basidiomycota are very high in snow‐covered soil (winter).

**Fig. S 2** Rarefaction plot of amplicon sequencing data from sites 0, 1, and 2. The vertical line is indicating the size of the 2^nd^ smallest sample, which was used as threshold for rarefying (= subsampling with replacement). The first two stages of SSD are saturated, but SSD 2 is not.

**Fig. S 3** Plot: Venn diagram based on amplicon sequencing data generated for the different stages of soil development in the sites 0, 1, and 2. The number of shared OTUs given separately for summer (left) and winter (right). Irrespective of season, most species are shared between the two later stages of soil development.
