# Supplementary material for: Emerging from the ice‐fungal communities are diverse and dynamic in earliest soil developmental stages of a receding glacier
Source: Environ Microbiol. 2019 Apr 11;21(5):1864–80. doi: 10.1111/1462-2920.14598 (PMC6849718; doi:10.1111/1462-2920.14598)
Supplement: Supplementary file 1 — Appendix S1: Supporting Information [file EMI-21-1864-s001.doc]

**Supporting Information**

**Table S1.** Physical and chemical soil parameters at the three earliest stages of soil development after de-glaciation. Pplant/Ptotal [%] is the proportion of plant-available phosphate in total phosphate. Data are given as means ± standard deviation; n = 7 for site 0, n = 8 for sites 1 and 2, respectively.

| Soil Parameter | Site 0  0-3 years | Site 1 9-14 years | Site 2 18-25 years |
| --- | --- | --- | --- |
| pH | 7.5 ± 0.1 | 7.5 ± 0.0 | 7.5 ± 0.0 |
| SOM [% DM] | 0.3 ± 0.3 | 0.6 ± 0.2 | 0.9 ± 0.3 |
| Ammonium [µg N · g^-1^ DM] | 0.03 ± 0.01 | 0.11 ± 0.14 | 0.11 ± 0.11 |
| Nitrate [µg N · g^-1^ DM] | 0.06 ± 0.02 | 0.30 ± 0.23 | 0.16 ± 0.16 |
| Plant-Available Phosphate [µg P · g^-1^ DM] | 0.21 ± 0.09 | 0.36 ± 0.08 | 0.39 ± 0.15 |
| Total Phosphate [µg P · g^-1^ DM] | 61.0 ± 7.2 | 85.3 ± 10.5 | 82.1 ± 18.4 |
| Pplant/Ptotal [% DM] | 0.34 ± 0.10 | 0.42 ± 0.06 | 0.49 ± 0.22 |
| Carbonate [% DM] | 2 - 10 | 2 - 25 | 10 - 25 |

SOM = soil organic matter; DM = soil dry matter; Pplant = plant-available phosphate; Ptotal = total phosphate

**Table S 2.** Matrix of Spearman correlation coefficients (r) between soil parameters at the three earliest stages of soil development after de-glaciation, n = 23. Significant correlations (p ˂ 0.05) are marked with an asterisk.

|  | SOM | Ammonium | Nitrate | Pplant | Ptotal | WC |
| --- | --- | --- | --- | --- | --- | --- |
| pH | -0.214 | 0.015 | 0.330 | -0.211 | 0.070 | 0.185 |
| SOM |  | 0.832* | 0.270 | 0.564* | 0.508* | 0.404 |
| Ammonium |  |  | 0.361 | 0.496* | 0.524* | 0.534* |
| Nitrate |  |  |  | 0.320 | 0.176 | 0.212 |
| Pplant |  |  |  |  | 0.696* | 0.141 |
| Ptotal |  |  |  |  |  | 0.237 |

SOM = soil organic matter; DM = soil dry matter; Pplant = plant-available phosphate; Ptotal = total phosphate; WC = water content

**Table S 3.** Fungal cOTUs (cultivated strains merged into operational taxonomic units based on a ITS1-rDNA-ITS2 sequence identity of 97%) isolated from in-growth mesh bags the three earliest stages of soil development after de-glaciation during the snow-free period (summer = S) and the period with snow-covered (winter = W). Site 0 is ice-free for 0-3 years, site 1 for 9-14 years, and site 2 for 18-25 years. GenBank accession numbers are provided for 99% sequence identity cOTUs clustering into one 97% sequence identity cOTU. Information on the ability to growth and 25°C, and on the growth form is also provided. **+** = all strains of one cOTU growth at 25 °C, **-** = no growth at 25 °C, **~**= cOTU contains strains, which grow at 25 °C. Filamentous fungi = f, dimorph fungi = d, yeast = y. Ascomycota = Asco, Basdiomycota = Basi, Mortierellomycotina = Mort, Mucoromycotina = Muco

| **ID** | **Identification** | **Phylum** | **Order** | **Genus** | **GenBank accession numbers** | | | **25°C** | **Growth** | | **W0** | | **W1** | | **W2** | | **S0** | | **S1** | | **S2** | |
| --- | --- | --- | --- | --- | --- | --- | --- | --- | --- | --- | --- | --- | --- | --- | --- | --- | --- | --- | --- | --- | --- | --- |
| cOTU59 | *Cladosporium* sp. | Asco | Capnodiales | *Cladosporium* | MF614981 | MF615037 | MF615039 | + | f | 1 | | 1 | | 1 | | 1 | | 1 | | 1 | |  |
| cOTU10 | *[Elasticomyces elasticus](https://blast.ncbi.nlm.nih.gov/Blast.cgi#alnHdr_1028916634)* | Asco | Capnodiales | *Elasticomyces* | MF614978 |  |  | - | f |  | |  | |  | | 1 | |  | |  | |  |
| cOTU18 | *[Ramularia](https://blast.ncbi.nlm.nih.gov/Blast.cgi#alnHdr_1097197552)* sp. | Asco | Capnodiales | *Ramularia* | MF614991 |  |  | - | f |  | |  | | 1 | |  | |  | |  | |  |
| cOTU26 | *[Penicillium](https://blast.ncbi.nlm.nih.gov/Blast.cgi#alnHdr_940377782)* sp. | Asco | Eurotiales | *Penicillium* | MF615005 |  |  | + | f | 1 | |  | |  | |  | |  | |  | |  |
| cOTU14 | *[Thysanophora penicillioides](https://blast.ncbi.nlm.nih.gov/Blast.cgi#alnHdr_74038641)* | Asco | Eurotiales | *Thysanophora* | MF614987 |  |  | + | f |  | |  | |  | | 1 | |  | |  | |  |
| cOTU50 | [*Botrytis cinerea*](https://unite.ut.ee/bl_forw.php?id=571050) | Asco | Helotiales | *Botrytis* | MF615031 |  |  | + | f |  | |  | |  | | 1 | |  | | 1 | |  |
| cOTU13 | *Chalara* sp. | Asco | Helotiales | [*Chalara*](https://www.ebi.ac.uk/ena/data/view/Taxon:Chalara) | MF614985 |  |  | + | f |  | | 1 | |  | |  | |  | |  | |  |
| cOTU36 | *[Cistella acuum](https://blast.ncbi.nlm.nih.gov/Blast.cgi#alnHdr_300862501)* | Asco | Helotiales | *Cistella* | MF615017 |  |  | + | f |  | |  | |  | |  | | 1 | |  | |  |
| cOTU22 | *[Gyoerffyella rcOTUla](https://blast.ncbi.nlm.nih.gov/Blast.cgi#alnHdr_1044656381)* | Asco | Helotiales | *Gyoerffyella* | MF615000 |  |  | - | f |  | |  | | 1 | |  | |  | |  | |  |
| cOTU103 | *Laetinaevia* sp. | Asco | Helotiales | *Laetinaevia* | MF615102 |  |  | - | f |  | |  | |  | |  | | 1 | |  | |  |
| cOTU21 | *Tetracladium* sp. | Asco | Helotiales | *Tetracladium* | MF614999 |  |  | - | f |  | |  | |  | |  | |  | | 1 | |  |
| cOTU23 | *Tetracladium* sp. | Asco | Helotiales | *Tetracladium* | MF615001 |  |  | + | f |  | |  | |  | |  | |  | | 1 | |  |
| cOTU27 | *Tetracladium* sp. | Asco | Helotiales | *Tetracladium* | MF614997 | MF615006 |  | ~ | f |  | |  | | 1 | |  | |  | | 1 | |  |
| cOTU57 | *Tetracladium* sp. | Asco | Helotiales | *Tetracladium* | MF614986 | MF615036 |  | ~ | f |  | |  | |  | | 1 | |  | |  | |  |
| cOTU100 | *Tetracladium* sp. | Asco | Helotiales | *Tetracladium* | MF614993 | MF615100 |  | + | f |  | |  | | 1 | |  | |  | | 1 | |  |
| cOTU52 | *Tetracladium* sp. | Asco | Helotiales | *Tetracladium* | MF614992 | MF615024 |  | - | f | 1 | |  | |  | |  | |  | |  | |  |
| cOTU56 | *Tetracladium* sp. | Asco | Helotiales | *Tetracladium* | MF614995 | MF615007 |  | + | f |  | |  | |  | |  | |  | | 1 | |  |
| cOTU43 | [Helotiales sp.](https://blast.ncbi.nlm.nih.gov/Blast.cgi#alnHdr_760236019) | Asco | Helotiales | unidentified | MF614975 | MF615023 |  | ~ | f | 1 | |  | | 1 | |  | |  | | 1 | |  |
| cOTU94 | Helotiales sp. | Asco | Helotiales | unidentified | MF615090 | MF615093 |  | + | f |  | |  | | 1 | | 1 | | 1 | | 1 | |  |
| cOTU24 | Helotiales sp. | Asco | Helotiales | unidentified | MF614994 | MF615003 |  | + | f |  | |  | | 1 | |  | | 1 | |  | |  |
| cOTU61 | Helotiales sp. | Asco | Helotiales | unidentified | MF615042 |  |  | - | f |  | |  | |  | | 1 | |  | |  | |  |
| cOTU53 | *Lecanicillium* sp. | Asco | Hypocreales | *Lecanicillium* | MF614969 | MF615033 |  | + | f | 1 | | 1 | |  | | 1 | |  | |  | |  |
| cOTU55 | *Neonectria* sp. | Asco | Hypocreales | *Neonectria* | MF615035 |  |  | + | f |  | |  | | 1 | |  | |  | | 1 | |  |
| cOTU31 | *[Paecilomyces anatarcticus](https://blast.ncbi.nlm.nih.gov/Blast.cgi#alnHdr_71608920)* | Asco | Hypocreales | *Paecilomyces* | MF615011 |  |  | - | f | 1 | |  | |  | | 1 | |  | |  | |  |
| cOTU72 | Hypocreales sp. | Asco | Hypocreales | *unidentified* | MF615059 |  |  | + | f | 1 | |  | |  | | 1 | |  | | 1 | |  |
| cOTU16 | *Oidiodendron* sp. | Asco | incertae sedis | *Oidiodendron* | MF614989 |  |  | - | f |  | |  | |  | | 1 | |  | |  | |  |
| cOTU102 | *Pseudogymnoascus* sp. | Asco | incertae sedis | *Pseudogymnoascus* | MF615046 | MF615047 | MF615101 | + | f | 1 | | 1 | |  | | 1 | |  | | 1 | |  |
| cOTU48 | Microascaceae sp. | Asco | Microascales | unidentified | MF615029 |  |  | - | f |  | |  | |  | |  | |  | | 1 | |  |
| cOTU20 | *Myrmecridium hiemale* | Asco | Myrmecridiales | *Myrmecridium* | MF614998 |  |  | + | f |  | |  | | 1 | |  | |  | |  | |  |
| cOTU42 | *[Alpinaria rhododendri](https://blast.ncbi.nlm.nih.gov/Blast.cgi#alnHdr_1196693180)* | Asco | Pleosporales | *Alpinaria* | MF614977 | MF614983 |  | - | f |  | |  | |  | |  | | 1 | |  | |  |
| cOTU34 | *Alternaria* sp. | Asco | Pleosporales | *Alternaria* | MF615015 |  |  | + | f |  | |  | | 1 | |  | |  | |  | |  |
| cOTU49 | *Alternaria* sp. | Asco | Pleosporales | *Alternaria* | MF615030 |  |  | + | f |  | |  | | 1 | |  | |  | |  | |  |
| cOTU11 | *[Epicoccum nigrum](https://blast.ncbi.nlm.nih.gov/Blast.cgi#alnHdr_1134784397)* | Asco | Pleosporales | *Epicoccum* | MF614980 |  |  | + | f | 1 | |  | |  | |  | |  | |  | |  |
| cOTU29 | *Leptosphaeria* sp. | Asco | Pleosporales | *Leptosphaeria* | MF615009 |  |  | + | f |  | |  | |  | | 1 | |  | |  | |  |
| cOTU64 | *Leptosphaeria* sp. | Asco | Pleosporales | *Leptosphaeria* | MF615051 |  |  | - | f | 1 | |  | | 1 | | 1 | |  | |  | |  |
| cOTU65 | *[Monodictys arctica](https://blast.ncbi.nlm.nih.gov/Blast.cgi#alnHdr_1044656433)* | Asco | Pleosporales | *Monodictys* | MF615013 | MF615052 |  | + | f |  | |  | | 1 | |  | | 1 | |  | |  |
| cOTU35 | *Periconia* sp. | Asco | Pleosporales | *Periconia* | MF615016 |  |  | + | f |  | |  | | 1 | |  | |  | |  | |  |
| cOTU38 | *Periconia* sp. | Asco | Pleosporales | *Periconia* | MF615019 |  |  | + | f |  | |  | | 1 | |  | |  | |  | |  |
| cOTU12 | *[Phoma herbarum](https://blast.ncbi.nlm.nih.gov/Blast.cgi#alnHdr_343131244)* | Asco | Pleosporales | *Phoma* | MF614982 |  |  | + | f |  | |  | |  | | 1 | |  | |  | |  |
| cOTU37 | Pleosporales sp. | Asco | Pleosporales | unidentified | MF615018 |  |  | - | f |  | |  | |  | |  | | 1 | |  | |  |
| cOTU80 | Pezizomycotina sp. | Asco | Sordariales | unidentified | MF615068 |  |  | + | f |  | |  | | 1 | | 1 | |  | |  | |  |
| cOTU33 | *Protomyces* sp. | Asco | Taphrinales | *Protomyces* | MF615014 |  |  | - | y |  | |  | | 1 | |  | |  | | 1 | |  |
| cOTU98 | *Protomyces* sp. | Asco | Taphrinales | *Protomyces* | MF615002 | MF615097 |  | - | y |  | |  | | 1 | |  | |  | | 1 | |  |
| cOTU82 | [Taphrinaceae sp.](https://unite.ut.ee/bl_forw.php?id=174998) | Asco | Taphrinales | unidentified | MF615070 |  |  | - | y |  | | 1 | | 1 | |  | | 1 | |  | |  |
| cOTU97 | Pezizomycotina sp. | Asco | unidentified | unidentified | MF614971 | MF614984 MF615085 | MF615079 MF615096 | ~ | f | 1 | |  | |  | | 1 | | 1 | |  | |  |
| cOTU3 | Asco sp. | Asco | unidentified | unidentified | MF614967 |  |  | + | f |  | |  | | 1 | |  | |  | |  | |  |
| cOTU44 | *Truncatella angustata* | Asco | Xylariales | *Truncatella* | MF615025 |  |  | + | f |  | |  | |  | |  | | 1 | |  | |  |
| cOTU39 | Xylariales sp. | Asco | Xylariales | unidentified | MF615020 |  |  | - | y |  | |  | |  | |  | | 1 | |  | |  |
| cOTU25 | Amphisphaeriaceae sp. | Asco | Xylariales | unidentified | MF615004 |  |  | - | f |  | |  | |  | | 1 | |  | |  | |  |
| cOTU76 | Agaricales sp. | Basi | Agaricales | unidentified | MF615063 |  |  | + | f |  | |  | |  | |  | | 1 | |  | |  |
| cOTU90 | Agaricales sp. | Basi | Agaricales | unidentified | MF615084 |  |  | + | f |  | |  | |  | | 1 | |  | |  | |  |
| cOTU54 | Agaricales sp. | Basi | Agaricales | unidentified | MF615034 |  |  | - | f | 1 | |  | |  | |  | | 1 | |  | |  |
| cOTU67 | Mrakiaceae sp. | Basi | Cystofilobasidiales | unidentified | MF615054 |  |  | - | y |  | |  | | 1 | |  | |  | |  | |  |
| cOTU89 | Mrakiaceae sp. | Basi | Cystofilobasidiales | unidentified | MF615083 |  |  | - | y | 1 | |  | |  | |  | |  | |  | |  |
| cOTU85 | Cystofilobasidiales sp. | Basi | Cystofilobasidiales | unidentified | MF615044 | MF615049 | MF615074 | - | y |  | |  | | 1 | |  | |  | | 1 | |  |
| cOTU73 | Entylomatales sp. | Basi | Entylomatales | *Entyloma* | MF615060 |  |  | + | d |  | |  | |  | | 1 | |  | |  | |  |
| cOTU75 | Entylomatales sp. | Basi | Entylomatales | *Entyloma* | MF615062 |  |  | + | d | 1 | |  | |  | | 1 | |  | |  | |  |
| cOTU101 | Filobasidiales sp. | Basi | Filobasidiales | unidentified | MF615094 | MF615098 |  | + | y |  | |  | |  | | 1 | |  | |  | |  |
| cOTU81 | Filobasidiales sp. | Basi | Filobasidiales | unidentified | MF615069 |  |  | - | y |  | | 1 | | 1 | |  | | 1 | | 1 | |  |
| cOTU83 | Filobasidiales sp. | Basi | Filobasidiales | unidentified | MF615071 |  |  | + | y |  | | 1 | |  | |  | | 1 | |  | |  |
| cOTU92 | [*Trichaptum* sp.](https://unite.ut.ee/bl_forw.php?id=583175) | Basi | Hymenochaetales | *Trichaptum* | MF615088 |  |  | + | f | 1 | |  | |  | |  | |  | |  | |  |
| cOTU78 | *Sampaiozyma* sp. | Basi | incertae sedis | *Sampaiozyma* | MF615065 |  |  | + | y |  | |  | | 1 | |  | |  | | 1 | |  |
| cOTU17 | *Glaciozyma watsonii* | Basi | Kriegeriales | *Glaciozyma* | MF614990 |  |  | - | y |  | |  | |  | | 1 | |  | |  | |  |
| cOTU45 | *Phenoliferia* sp. | Basi | Kriegeriales | *Phenoliferia* | MF615026 |  |  | - | y | 1 | |  | |  | | 1 | |  | |  | |  |
| cOTU47 | *Phenoliferia* sp. | Basi | Kriegeriales | *Phenoliferia* | MF615028 |  |  | - | y |  | |  | |  | | 1 | |  | |  | |  |
| cOTU104 | Phenoliferia sp. | Basi | Kriegeriales | Phenoliferia | MF615078 | MF615103 |  | - | y | 1 | |  | |  | | 1 | |  | |  | |  |
| cOTU105 | Phenoliferia sp. | Basi | Kriegeriales | Phenoliferia | MF614979 | MF615077 MF615081 | MF615080 MF615087 | - | f | 1 | | 1 | |  | | 1 | |  | |  | |  |
| cOTU69 | *Heterobasidion* sp. | Basi | Russulales | *Heterobasidion* | MF615056 |  |  | + | f | 1 | |  | |  | | 1 | |  | |  | |  |
| cOTU71 | Russulales sp. | Basi | Russulales | unidentified | MF615058 |  |  | + | f |  | |  | | 1 | |  | |  | |  | |  |
| cOTU46 | [Sebacinaceae](https://unite.ut.ee/bl_forw.php?id=14513) | Basi | Sebacinales | unidentified | MF615027 |  |  | - | f |  | |  | |  | |  | | 1 | |  | |  |
| cOTU7 | [*Rhodotorula* sp.](https://unite.ut.ee/bl_forw.php?id=309922) | Basi | Sporidiobolales | *Rhodotorula* | MF614973 |  |  | - | y | 1 | |  | |  | |  | |  | |  | |  |
| cOTU51 | *Sporidiobolus* sp. | Basi | Sporidiobolales | *Sporidiobolus* | MF615032 |  |  | + | y | 1 | |  | |  | |  | |  | |  | |  |
| cOTU79 | Sporidiobolales sp. | Basi | Sporidiobolales | unidentified | MF615067 |  |  | - | y |  | |  | |  | |  | |  | | 1 | |  |
| cOTU93 | Sporidiobolales sp. | Basi | Sporidiobolales | unidentified | MF615050 | MF615089 |  | - | y |  | |  | |  | | 1 | |  | |  | |  |
| cOTU6 | *Dioszegia fristingensis* | Basi | Tremellales | *Dioszegia* | MF614972 |  |  | - | y |  | |  | | 1 | |  | |  | | 1 | |  |
| cOTU32 | *Dioszegia hungarica* | Basi | Tremellales | *Dioszegia* | MF615012 |  |  | + | y |  | | 1 | | 1 | |  | | 1 | | 1 | |  |
| cOTU5 | *Phaeotremella skinneri* | Basi | Tremellales | *Phaeotremella* | MF614970 |  |  | + | y |  | |  | |  | | 1 | |  | |  | |  |
| cOTU8 | *[Vishniacozyma carnescens](https://blast.ncbi.nlm.nih.gov/Blast.cgi#alnHdr_1035489901)* | Basi | Tremellales | *Vishniacozyma* | MF614974 |  |  | + | y |  | |  | |  | | 1 | |  | |  | |  |
| cOTU88 | Tremellomycetes sp. | Basi | Tremellales | *Vishniacozyma* | MF615038 | MF615082 |  | + | y |  | |  | | 1 | | 1 | | 1 | | 1 | |  |
| cOTU28 | [Microbotryomycetes sp.](https://unite.ut.ee/bl_forw.php?id=406303) | Basi | unidentified | unidentified | MF615008 |  |  | - | y |  | |  | | 1 | |  | |  | |  | |  |
| cOTU58 | *Mortierella* sp. | Mort | Mortierellales | *Mortierella* | MF615040 |  |  | - | f | 1 | |  | |  | |  | |  | |  | |  |
| cOTU70 | *Mortierella* sp. | Mort | Mortierellales | *Mortierella* | MF615057 |  |  | + | f |  | |  | | 1 | |  | |  | | 1 | |  |
| cOTU87 | *[Mortierella](https://blast.ncbi.nlm.nih.gov/Blast.cgi#alnHdr_829582490)* sp. | Mort | Mortierellales | *Mortierella* | MF615048 | MF615072 | MF615075 | - | f | 1 | | 1 | |  | |  | |  | |  | |  |
| cOTU95 | *[Mortierella](https://blast.ncbi.nlm.nih.gov/Blast.cgi#alnHdr_829582490)* sp. | Mort | Mortierellales | *Mortierella* | MF615066 | MF615092 |  | + | f | 1 | | 1 | | 1 | | 1 | | 1 | | 1 | |  |
| cOTU96 | *[Mortierella](https://blast.ncbi.nlm.nih.gov/Blast.cgi#alnHdr_829582490)* sp. | Mort | Mortierellales | *Mortierella* | MF615091 | MF615095 |  | + | f |  | |  | |  | | 1 | |  | |  | |  |
| cOTU99 | *[Mortierella](https://blast.ncbi.nlm.nih.gov/Blast.cgi#alnHdr_829582490)* sp. | Mort | Mortierellales | *Mortierella* | MF615099 |  |  | + | f | 1 | | 1 | | 1 | |  | | 1 | | 1 | |  |
| cOTU1 | *Mucor* *flavus* | Muco | Mucorales | *Mucor* | MF614965 |  |  | + | f |  | |  | |  | | 1 | |  | |  | |  |
| cOTU2 | *Mucor* *flavus* | Muco | Mucorales | *Mucor* | MF614966 |  |  | + | f |  | |  | |  | | 1 | |  | |  | |  |
| cOTU84 | *Mucor* *hiemalis* | Muco | Mucorales | *Mucor* | MF615073 |  |  | + | f | 1 | |  | | 1 | |  | |  | |  | |  |
| cOTU86 | *Mucor* *hiemalis* | Muco | Mucorales | *Mucor* | MF615076 |  |  | + | y | 1 | |  | | 1 | |  | |  | | 1 | |  |
| cOTU77 | *Mucor microsporus* | Muco | Mucorales | *Mucor* | MF615064 |  |  | + | f |  | |  | | 1 | |  | |  | |  | |  |

**Table S 4**. Differences in fungal community composition detected by amplicon sequencing among the following *a priori* defined groups: in-growth mesh bags (MBs), soil samples with snow cover (soil snow), bare soil samples two weeks after snow-melt (soil bare), stages of soil development (SSDs = sites 0, 1, 2) sampling season (summer, winter). Significant differences are assessed by analysis of similarity (ANOSIM) plus effect sizes, and explained variances of factors assessed by permutational multivariate analysis of variance (PERMANOVA) for the individual data subsets.

| Comparison | Data Subset | ANOSIM – R | PERMANOVA - F | PERMANOVA – R^2^ |
| --- | --- | --- | --- | --- |
| Succession:  sites 0 vs. 1 vs. 2 | MBs | 0.781*** | 5.74*** | 0.29 |
| Seasonality:  summer vs. winter | MBs | 0.103* | 2.28* | 0.06 |
| Method:  MBs vs. soil | Site 2 with snow | -0.176 | 0.66 | 0.08 |
| Dynamics of change: snow covered vs. 2-week snow-free | Site 2 soil samples | 0.006 | 0.83 | 0.11 |

* = P < 0.05; *** = P < 0.001; R = ANOSIM test statistic; F = F-ratio of PERMANOVA; R^2^ = variance estimator

**Table S 5**. Comparison of amplicon sequencing data with sequences generated from fungi isolates from the same MBs. Local Blast of cOTU (ITS2-extracted) against NGS-based OTUs.

| | **taxonomy of cultivation-based OTUs** | **taxonomy of NGS-based OTUs** | | --- | --- | | cOTU096 s_unidentified_Mortierella | OTU80 g_Mortierella | | cOTU097 s_unidentified_Phialophora | OTU790 g_Phialophora | | cOTU094 s_unidentified_Pezizella | OTU759 g_Tetracladium | | cOTU095 s_unidentified_Mortierella | OTU40 s_Mortierella_alpina | | cOTU092 s_unidentified_Trichaptum | OTU388 s_Trichaptum_abietinum | | cOTU093 s_unidentified_Sporidiobolales | OTU185 o_Sporidiobolales | | cOTU090 s_unidentified_Hypholoma | NA | | cOTU098 s_Protomyces_inouyei | NA | | cOTU099 s_unidentified_Mortierella | OTU35 g_Mortierella | | cOTU052 s_unidentified_Tetracladium | OTU952 g_Tetracladium | | cOTU053 s_Lecanicillium_saksenae | NA | | cOTU050 s_Botrytis_cinerea | OTU867 g_Botrytis | | cOTU051 s_unidentified_Sporidiobolus | NA | | cOTU056 s_unidentified_Tetracladium | OTU949 g_Tetracladium | | cOTU057 s_unidentified_Tetracladium | OTU794 g_Tetracladium | | cOTU054 s_unidentified_Psilocybe | OTU510 o_Agaricales | | cOTU055 s_Neonectria_candida | OTU657 g_Neonectria | | cOTU058 s_unidentified_Mortierella | OTU80 g_Mortierella | | cOTU059 s_unidentified_Cladosporium | OTU786 g_Cladosporium | | cOTU018 s_unidentified_Ramularia | OTU978 g_Ramularia | | cOTU016 s_unidentified_Oidiodendron | NA | | cOTU017 s_Glaciozyma_watsonii | OTU204 c_Microbotryomycetes | | cOTU014 s_Thysanophora_penicillioides | NA | | cOTU012 s_Phoma_herbarum | OTU682 g_Epicoccum | | cOTU013 s_Chalara_holubovae | OTU769 o_Helotiales | | cOTU010 s_Elasticomyces_elasticus | NA | | cOTU011 s_Epicoccum_nigrum | OTU682 g_Epicoccum | | cOTU069 s_unidentified_Heterobasidion | OTU410 g_Heterobasidion | | cOTU027 s_unidentified_Tetracladium | OTU904 g_Tetracladium | | cOTU026 s_Penicillium_thomii | NA | | cOTU025 s_unidentified_Seimatosporium | OTU711 f_Amphisphaeriaceae | | cOTU024 s_unidentified_Helotiales | OTU1038 o_Helotiales | | cOTU023 s_unidentified_Tetracladium | OTU849 o_Helotiales | | cOTU022 s_Gyoerffyella_rotula | OTU890 o_Helotiales | | cOTU021 s_unidentified_Tetracladium | OTU883 g_Tetracladium | | cOTU020 s_Myrmecridium_hiemale | NA | | cOTU075 s_unidentified_Entylomatales | NA | | cOTU061 s_unidentified_Helotiales | OTU769 o_Helotiales | | cOTU067 s_unidentified_Mrakia | OTU150 g_Mrakiella | | cOTU029 s_Leptosphaeria_sclerotioides | OTU676 g_Leptosphaeria | | cOTU028 s_unidentified_Microbotryomycetes | NA | | cOTU005 s_Phaeotremella_skinneri | NA | | cOTU006 s_Dioszegia_fristingensis | OTU1027 s_Dioszegia_fristingensis | | cOTU007 s_Rhodotorula_glacialis | OTU297 c_Microbotryomycetes | | cOTU003 s_unidentified_Dothideomycetes | NA | | cOTU008 s_Cryptococcus_carnescens | OTU1004 g_Vishniacozyma | | cOTU103 s_unidentified_Laetinaevia | OTU759 g_Tetracladium | | cOTU102 s_Pseudogymnoascus_destructans | OTU832 g_Pseudogymnoascus | | cOTU101 s_unidentified_Goffeauzyma | OTU162 c_Tremellomycetes | | cOTU100 s_unidentified_Tetracladium | OTU905 g_Tetracladium | | cOTU105 s_Phenoliferia_psychrophenolica | OTU363 g_Rhodotorula | | cOTU104 s_Phenoliferia_glacialis | OTU363 g_Rhodotorula | | cOTU065 s_Monodictys_arctica | OTU729 g_Leptosphaeria | | cOTU064 s_Leptosphaeria_sclerotioides | OTU676 g_Leptosphaeria | | cOTU034 s_Alternaria_alternata | OTU617 g_Alternaria | | cOTU035 s_Periconia_digitata | NA | | cOTU036 s_Cistella_acuum | OTU769 o_Helotiales | | cOTU037 s_unidentified_Pleosporales | OTU744 o_Pleosporales | | cOTU031 s_Paecilomyces_antarcticus | NA | | cOTU032 s_Dioszegia_hungarica | OTU976 s_Dioszegia_hungarica | | cOTU033 s_Protomyces_inouyei | NA | | cOTU070 s_unidentified_Mortierella | OTU63 s_Mortierella_exigua | | cOTU071 s_unidentified_Peniophora | NA | | cOTU072 s_unidentified_Paecilomyces | NA | | cOTU073 s_unidentified_Entylomatales | OTU93 g_Entyloma | | cOTU038 s_Periconia_byssoides | OTU702 g_Pithomyces | | cOTU039 s_unidentified_Phyllachorales | OTU561 p_Asco | | cOTU076 s_unidentified_Hypholoma | NA | | cOTU077 s_Mucor_microsporus | NA | | cOTU078 s_unidentified_Sampaiozyma | OTU318 c_Microbotryomycetes | | cOTU079 s_unidentified_Sporidiobolales | OTU185 o_Sporidiobolales | | cOTU089 s_unidentified_Mrakia | OTU150 g_Mrakiella | | cOTU088 s_unidentified_Vishniacozyma | OTU1004 g_Vishniacozyma | | cOTU047 s_Phenoliferia_himalayensis | OTU278 c_Microbotryomycetes | | cOTU046 s_unidentified_Sebacinaceae | OTU423 c_Agaricomycetes | | cOTU043 s_unidentified_Mycoarthris | OTU928 g_Mycoarthris | | cOTU042 s_Alpinaria_rhododendri | OTU771 g_Herpotrichia | | cOTU081 s_unidentified_Piskurozyma | OTU170 g_Filobasidium | | cOTU080 s_unidentified_Soradiales | OTU875 g_Cadophora | | cOTU083 s_unidentified_Piskurozyma | OTU189 g_Cryptococcus | | cOTU082 s_unidentified_Taphrinaceae | OTU374 k_Fungi | | cOTU085 s_unidentified_Mrakia | OTU150 g_Mrakiella | | cOTU084 s_Mucor_hiemalis | NA | | cOTU087 s_unidentified_Mortierella | OTU80 g_Mortierella | | cOTU086 s_Mucor_hiemalis | NA | | cOTU045 s_Phenoliferia_psychrophenolica | OTU363 g_Rhodotorula | | cOTU048 s_unidentified_Wardomyces | NA | | cOTU044 s_Truncatella_angustata | NA | | cOTU049 s_Alternaria_penicillata | OTU618 g_Alternaria | |  |  |  |
| --- | --- | --- | --- | --- | --- | --- | --- | --- | --- | --- | --- | --- | --- | --- | --- | --- | --- | --- | --- | --- | --- | --- | --- | --- | --- | --- | --- | --- | --- | --- | --- | --- | --- | --- | --- | --- | --- | --- | --- | --- | --- | --- | --- | --- | --- | --- | --- | --- | --- | --- | --- | --- | --- | --- | --- | --- | --- | --- | --- | --- | --- | --- | --- | --- | --- | --- | --- | --- | --- | --- | --- | --- | --- | --- | --- | --- | --- | --- | --- | --- | --- | --- | --- | --- | --- | --- | --- | --- | --- | --- | --- | --- | --- | --- | --- | --- | --- | --- | --- | --- | --- | --- | --- | --- | --- | --- | --- | --- | --- | --- | --- | --- | --- | --- | --- | --- | --- | --- | --- | --- | --- | --- | --- | --- | --- | --- | --- | --- | --- | --- | --- | --- | --- | --- | --- | --- | --- | --- | --- | --- | --- | --- | --- | --- | --- | --- | --- | --- | --- | --- | --- | --- | --- | --- | --- | --- | --- | --- | --- | --- | --- | --- | --- | --- | --- | --- | --- | --- | --- | --- | --- | --- | --- | --- | --- | --- | --- | --- | --- | --- | --- | --- | --- |

Table S 5. Additional Packages used for analyses carried out in R (3.3.2)

attached base packages: stats, graphics, grDevices, utils, datasets, methods, base;

other attached packages: scales_1.0.0, ggsci_2.9, ggpubr_0.1.8, magrittr_1.5, tidyr_0.8.1, multcomp_1.4-8, TH.data_1.0-9, MASS_7.3-50, survival_2.42-6, mvtnorm_1.0-8, gridExtra_2.3, RColorBrewer_1.1-2, vegan_2.5-2, lattice_0.20-35, permute_0.9-4, ggplot2_3.0.0, phyloseq_1.22.3;

loaded via a namespace (and not attached): zoo_1.8-3, tidyselect_0.2.4, reshape2_1.4.3, purrr_0.2.5, splines_3.4.4, rhdf5_2.22.0, colorspace_1.3-2, stats4_3.4.4, mgcv_1.8-24, rlang_0.2.2, pillar_1.3.0, glue_1.3.0, withr_2.1.2, BiocGenerics_0.24.0, bindrcpp_0.2.2, foreach_1.4.4, plyr_1.8.4, bindr_0.1.1, stringr_1.3.1, zlibbioc_1.24.0, Biostrings_2.46.0, munsell_0.5.0, gtable_0.2.0, codetools_0.2-15, labeling_0.3, Biobase_2.38.0, IRanges_2.12.0, biomformat_1.7.0, parallel_3.4.4, Rcpp_0.12.18, S4Vectors_0.16.0, jsonlite_1.5, XVector_0.18.0, digest_0.6.16, stringi_1.1.7, dplyr_0.7.6, grid_3.4.4, ade4_1.7-13, tools_3.4.4, sandwich_2.5-0, lazyeval_0.2.1, tibble_1.4.2, cluster_2.0.7-1, crayon_1.3.4, ape_5.1, pkgconfig_2.0.2, Matrix_1.2-14, data.table_1.11.4, assertthat_0.2.0, rstudioapi_0.7, iterators_1.0.10, R6_2.2.2, multtest_2.34.0, igraph_1.2.2, nlme_3.1-137, compiler_3.4.4,

🡪 place Fig. S 1.

Fig. S 1. Relative seasonal abundances of fungal phyla occurring in earliest stages of soil development ranging from 0 to 25 years (site 0, site 1 and site 2) based on amplicon sequencing data. Ascomycota dominate during early soil development. But especially during the first two stages of soil development, relative abundances of Basidiomycota are very high in snow-covered soil (winter).

🡪 place Fig. S 2.

Fig. S 2. Rarefaction plot of amplicon sequencing data from sites 0, 1, and 2. The vertical line is indicating the size of the 2^nd^ smallest sample, which was used as threshold for rarefying (= subsampling with replacement). The first two stages of SSD are saturated, but SSD 2 is not.

🡪 place Fig. S 3.

Fig. S 3. Plot: Venn diagram based on amplicon sequencing data generated for the different stages of soil development in the sites 0, 1, and 2. The number of shared OTUs given separately for summer (left) and winter (right). Irrespective of season, most species are shared between the two later stages of soil development.
