# Supplementary figures and images for: Emerging from the ice‐fungal communities are diverse and dynamic in earliest soil developmental stages of a receding glacier
Source: Environ Microbiol. 2019 Apr 11;21(5):1864–80. doi: 10.1111/1462-2920.14598 (PMC6849718; doi:10.1111/1462-2920.14598)

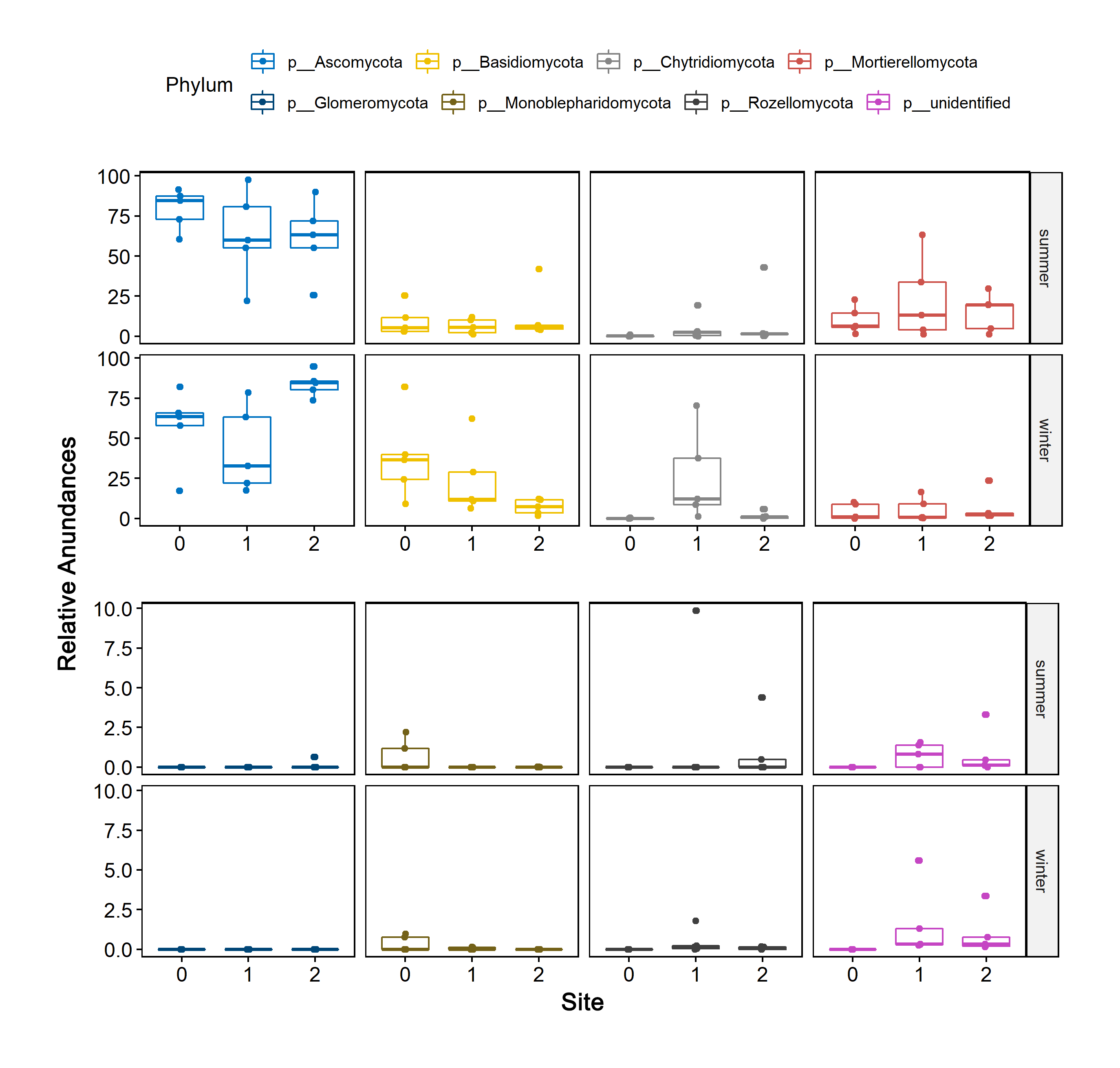

Supplement: Supplementary file 2 — Fig. S 1 Relative seasonal abundances of fungal phyla occurring in earliest stages of soil development ranging from 0 to 25 years (site 0, site 1 and site 2) based on amplicon sequencing data. Ascomycota dominate during early soil development. But especially during the first two stages of soil development, relative abundances of Basidiomycota are very high in snow‐covered soil (winter). [file EMI-21-1864-s002.tif]

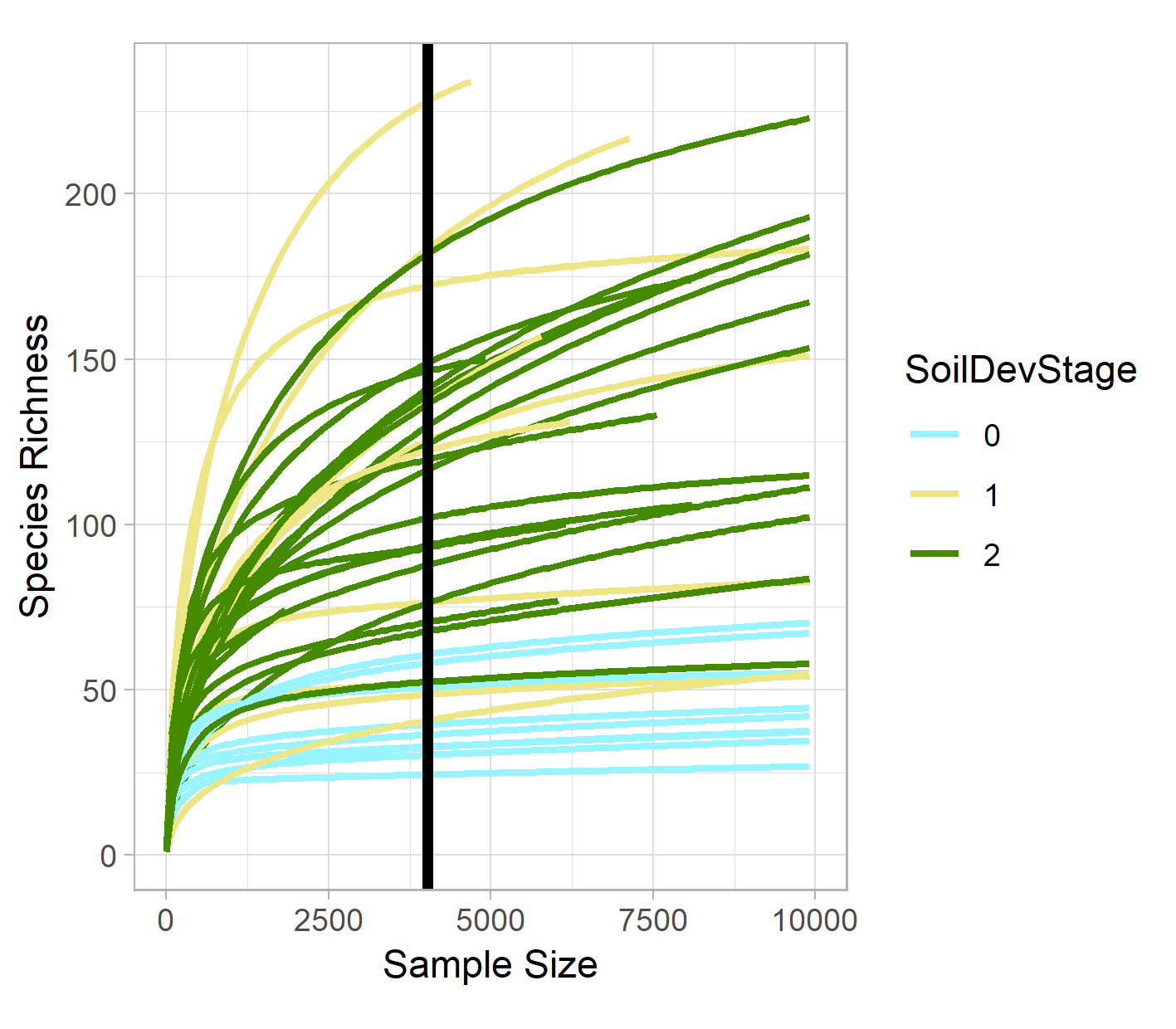

Supplement: Supplementary file 3 — Fig. S 2 Rarefaction plot of amplicon sequencing data from sites 0, 1, and 2. The vertical line is indicating the size of the 2nd smallest sample, which was used as threshold for rarefying (= subsampling with replacement). The first two stages of SSD are saturated, but SSD 2 is not. [file EMI-21-1864-s003.tif]

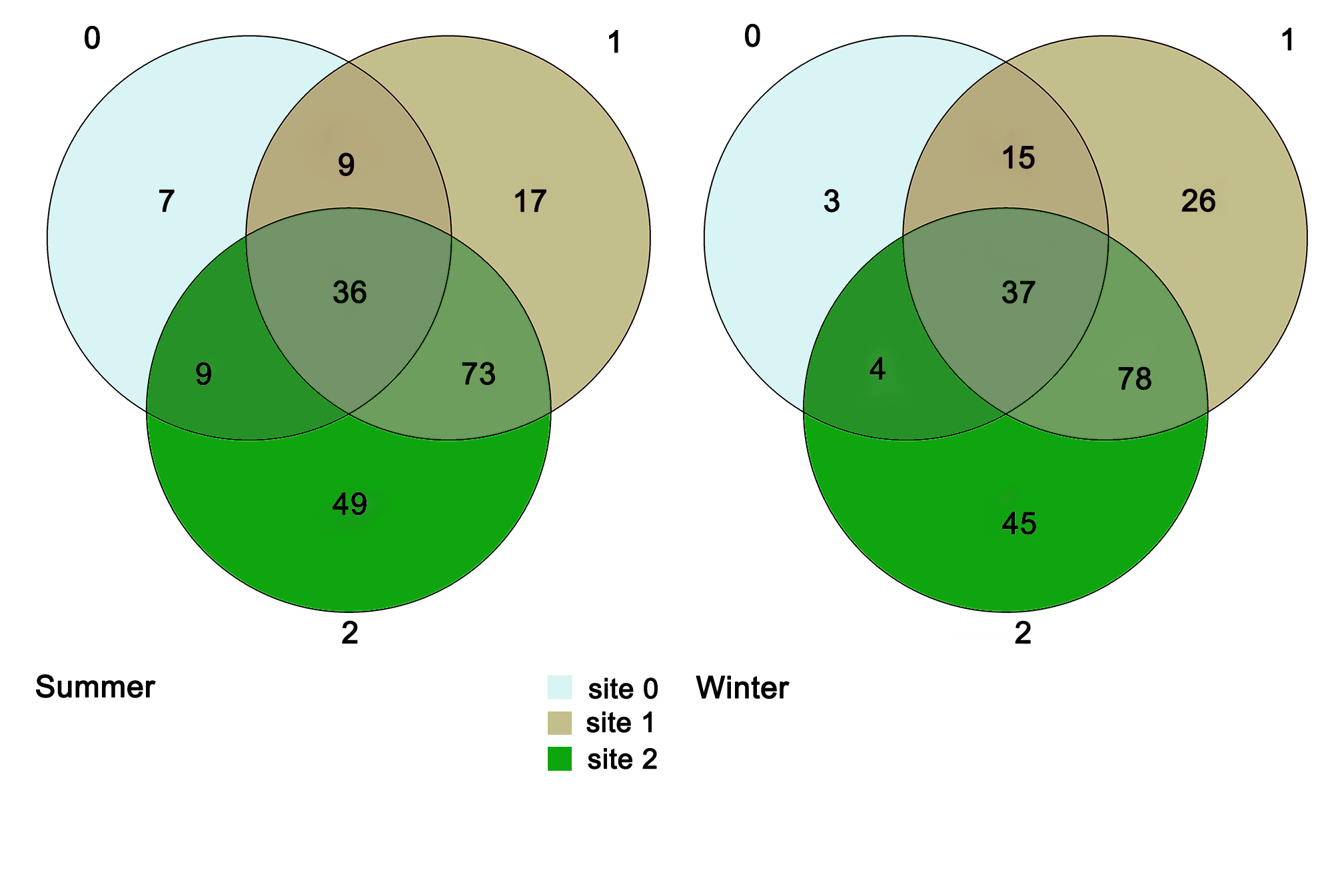

Supplement: Supplementary file 4 — Fig. S 3 Plot: Venn diagram based on amplicon sequencing data generated for the different stages of soil development in the sites 0, 1, and 2. The number of shared OTUs given separately for summer (left) and winter (right). Irrespective of season, most species are shared between the two later stages of soil development. [file EMI-21-1864-s004.tif]
